# Renalase Challenges the Oxidative Stress and Fibroproliferative Response in COVID-19

**DOI:** 10.1155/2022/4032704

**Published:** 2022-09-12

**Authors:** Dijana Stojanovic, Miodrag Stojanovic, Jelena Milenkovic, Aleksandra Velickov, Aleksandra Ignjatovic, Maja Milojkovic

**Affiliations:** ^1^Department of Pathophysiology, Faculty of Medicine, University of Nis, Nis, Serbia; ^2^Department of Medical Statistics and Informatics, Faculty of Medicine, University of Nis, Nis, Serbia; ^3^Center of Informatics and Biostatistics in Healthcare, Institute for Public Health, Nis, Serbia; ^4^Department of Histology and Embryology, Faculty of Medicine, University of Nis, Nis, Serbia

## Abstract

The hallmark of the coronavirus disease 2019 (COVID-19) pathophysiology was reported to be an inappropriate and uncontrolled immune response, evidenced by activated macrophages, and a robust surge of proinflammatory cytokines, followed by the release of reactive oxygen species, that synergistically result in acute respiratory distress syndrome, fibroproliferative lung response, and possibly even death. For these reasons, all identified risk factors and pathophysiological processes of COVID-19, which are feasible for the prevention and treatment, should be addressed in a timely manner. Accordingly, the evolving anti-inflammatory and antifibrotic therapy for severe COVID-19 and hindering post-COVID-19 fibrosis development should be comprehensively investigated. Experimental evidence indicates that renalase, a novel amino-oxidase, derived from the kidneys, exhibits remarkable organ protection, robustly addressing the most powerful pathways of cell trauma: inflammation and oxidative stress, necrosis, and apoptosis. As demonstrated, systemic renalase administration also significantly alleviates experimentally induced organ fibrosis and prevents adverse remodeling. The recognition that renalase exerts cytoprotection *via* sirtuins activation, by raising their NAD^+^ levels, provides a “proof of principle” for renalase being a biologically impressive molecule that favors cell protection and survival and maybe involved in the pathogenesis of COVID-19. This premise supports the rationale that renalase's timely supplementation may prove valuable for pathologic conditions, such as cytokine storm and related acute respiratory distress syndrome. Therefore, the aim for this review is to acknowledge the scientific rationale for renalase employment in the experimental model of COVID-19, targeting the acute phase mechanisms and halting fibrosis progression, based on its proposed molecular pathways. Novel therapies for COVID-19 seek to exploit renalase's multiple and distinctive cytoprotective mechanisms; therefore, this review should be acknowledged as the thorough groundwork for subsequent research of renalase's employment in the experimental models of COVID-19.

## 1. Introduction

The coronavirus disease 2019 (COVID-19), from its outbreak in China, in December 2019, until May 2022, according to the World Health Organization (WHO) [1] has globally affected more than 526 million people, of whom more than 6.28 million have died. Moreover, according to the Centers for Disease Control and Prevention (CDC) data, [2] in the USA, particularly, more than 83.7 million COVID-19 cases have been confirmed, with total death cases reported to be more than 1.1 million. Such horrifying evidence categorizes the COVID-19 pandemic to be the most unprecedented global health-care crisis in the modern era, whose mid- and long-term complications are yet to be evaluated. Nonetheless, according to the number of COVID-19 affected individuals, all manner of consequences present a hitherto unseen burden upon the health-care system in general.

At the time of writing, expertise on COVID-19 advances daily, and the global vaccination against severe acute respiratory syndrome coronavirus 2 (SARS-CoV-2) infection, has begun to be widely accepted [2]. However, despite these achievements, certain concerns indisputably remain. Notably, few significant variants have emerged within the SARS-CoV-2 genome, which may increase the virus' transmissibility, resulting in a prompt replacement of the current lineages [3, 4]. Therefore, apprehension remains as to whether these viral variants may increase the possibilities of reinfections or may generate vaccine failure, allowing the pandemic to expand. Secondly, SARS-CoV-2 infection is commonly asymptomatic, particularly for the younger age group, being contagious before the onset of symptoms [5], referred to as the virus's particularity that widely contributes to its spread [6]. Additionally, there is a significant and substantial possibility that the evolution of SARS and other novel viruses persists [3]; therefore, a thorough quest for an appropriate therapy should be maintained, regardless of vaccine development. It will be challenging to widen the existing protocols and to employ some new agents to protect and effectively treat COVID-19 and familiar diseases.

Nevertheless, as explained elsewhere, COVID-19 clinical presentations may be asymptomatic or mild cases [1–5], whereas the major cause of COVID-19 morbidity and mortality was identified to be the cytokine storm and its associated acute respiratory distress syndrome (ARDS) [7–14]. The phenomenon “cytokine storm” refers to an uncontrolled systemic inflammatory response, generating great amounts of proinflammatory cytokines and chemokines [7–14], followed by a fibroproliferative drive in the lungs, that is likely to progress into pulmonary fibrosis [12, 13, 15–17], with substantial fibrotic consequences.

Additionally, a novel protein, renalase, was identified. The most recent evidence of renalase's cellular mechanisms, pathophysiology, possible therapeutic validity and its experimentally (*in vivo* and *in vitro*) demonstrated pleiotropy to rapidly and effectively alleviate inflammation, oxidative stress, apoptosis, necrosis, and organ fibrosis [18–43], supports the hypothesis for renalase to become, at least to some extent, a brand-new therapeutic molecule in the course of the SARS-CoV-2 related disease.

In this review, our aim is to acknowledge a comprehensive overview of the pertinent evidence on renalase, regarding its powerful anti-inflammatory, antioxidative, antiapoptotic, and antifibrotic properties within the context of the current COVID-19 pandemic. We, however, aim to provide a scientific rationale for renalase employment in the experimental model of COVID-19, to target the acute phase mechanisms and halt fibrosis progression, based on its proposed molecular pathways. Moreover, the compiled data herein may be used as an accurate reference for renalase pathways so far identified, and may presumably be of value for subsequent experimental research design, regarding renalase's therapeutic potential in SARS-CoV-2-related infection. Therefore, we hypothesized that renalase administration, synergistically employed with other approved agents, may be an appealing strategy for challenging the cytokine storm consequences and fibroproliferative drive in the lungs of COVID-19-infected individuals. To the extent of our knowledge, these findings represent some original perspectives regarding renalase analysis in the context of the current pandemic. Initially, we will briefly discuss the rationale for antifibrotic therapy in patients subject to COVID-19.

We searched PubMed for manuscripts published in English between January 2021 and May 2022, using combinations of the following keywords: renalase, inflammation, NAD+, SARS, SARS-CoV-2, COVID-19, cytokine storm, sirtuins, autophagy, ARDS, fibroproliferative response, and pulmonary fibrosis. However, it has to be recognized that a vastly growing library of literature in this field has been published and is changing at great speed.

## 2. Scientific Rationale for Antifibrotic Therapy in COVID-19

The most prevalent reason for hospital treatment of COVID-19 patients was reported to be the development of interstitial pneumonia that is likely to progress into COVID-19-related acute respiratory distress syndrome and that has been documented, according to the design of the study, in 15% [44], or even in 40% of COVID-19 patients, whereas 20% of ARDS presented in its most severe and life-threatening forms [45].

The majority of patients with COVID-19-related ARDS survive the acute phase of the disease; however, their outcomes, in relation to the development of long term complications, have been inconclusive, and speculative so far, and require adequate future studies [46]. In line with this, evidence shows that a considerable proportion of ARDS survivors, regardless of the underlying cause, confronts serious complications and dies from progressive pulmonary fibrosis [47]. Fibroproliferative response in COVID-19 patients is an expected consequence, and may be anticipated based on several meaningful premises, that will be briefly discussed below. Regarding the number of infected individuals so far, particularly those treated with invasive ventilation, it is feasible to expect a substantial burden of post-COVID pulmonary fibrosis [46].

Notably, at the very beginning of the pandemic, the initial small case series demonstrated that unique pathological findings described in patients with COVID-19 included exudative diffuse alveolar damage with massive capillary congestion, followed by the formation of multiple thrombi [48–50]. However, more comprehensive research [51] revealed that the majority of post-mortem analyses documented exudative and early or intermediate proliferative phases of diffuse alveolar damage. This was coupled with findings characteristic of interstitial pneumonia, organizing pneumonia and acute fibrinous organizing pneumonia, with alveolar spaces populated with granulocytes and fibrin. Inflammatory infiltrate was verified in all cases and included heavy alveolar macrophage accumulation and interstitial lymphocyte infiltration. The fibrotic phase of diffuse alveolar damage was, however, documented only as focally distributed, implying that patients did not progress to the fibrotic phase, most likely owing to their death. These findings may be discussed with regard to the previous evidence that the early phase of ARDS (within 24 h of the onset of COVID-19 symptoms), regardless of its etiology, is associated with substantial fibroproliferation, supporting the theory that profibrotic mechanisms in the setting of ARDS are promptly upregulated, and that fibrosis begins early in the course of ARDS [15, 16].

Moreover, the comparative analysis of typical ARDS and COVID-19-associated ARDS revealed that, even diffuse alveolar damage was a prevailing pathological finding in both syndromes [52], fibrosis or fibrous stripes were more common in CT scans of COVID-19 patients [53, 54], possibly implying a more complex pathophysiology of a new coronavirus disease, particularly when conjoined with the scrutiny of sizable microvascular thrombosis [12, 13, 48–53]. The fibrous tissue may be regarded as a good prognostic factor for COVID-19 patients, indicating disease resolution [55]. However, it is more likely that fibrosis development should be recognized as a factor indicating a worse outcome of COVID-19, considering that it will eventually progress to fibrotic interstitial lung disease [56]. Given the heterogeneity of pulmonary alteration in COVID-19, it was documented that CT scan findings may significantly differ among the affected patients, owing to the identification of few distinct phenotypes of COVID-19-related ARDS, whereas at least one phenotype will, eventually, result in the development of fibrosis [57].

Secondly, the recognition that SARS-CoV-2 and SARS-CoV viruses share substantial resemblances (79.5% sequence identity) [3, 4, 9] may indicate that similar long-term complications evidenced after SARS-CoV infection may also be an important issue in COVID-19 survival. Therefore, documented chronic sequalae, such as fibrotic pulmonary remodeling and restrictive lung abnormalities, associated with impaired exercise tolerance and poor quality of life in patients who survived the SARS-CoV infection are likely to be an issue in COVID-19, as well [58]. The reports show that biopsy findings, performed just a few weeks following the onset of symptoms in SARS-CoV infection, revealed a significant accumulation of inflammatory cells, coupled with fibrin and fibroblasts, undoubtedly suggesting an initial fibrosis development [58]. Observation of evident reticular changes in pulmonary tissue in patients with SARS, immediately after the second week of the onset of symptoms, which will likely persist in half of all patients, beyond 4 weeks, raised the necessity for long-term follow-ups, in order to comprehensively establish whether observed reticulation represents irreversible fibrosis [59]. Therefore, it is feasible to expect the same issues in COVID-19 patients. A one-year follow-up documented that 27.8% of SARS-CoV convalescents had abnormal chest radiography findings, which correlated with the degree of functional impairment, along with significantly lower exercise capacity and health status [60]. Accordingly, two-year follow-up [61] evidenced persistent deterioration of lung function in SARS survivors, along with impaired exercise capacity and health status. The most recent report on SARS patients, which completed the 15-year follow-up, revealed residual lung diffusion abnormalities in one-third of the participants, while 4.6% of them presented with residual interstitial lesions [62]. Convincing evidence now exists that SARS-CoV patients (and presumably COVID-19) experience pulmonary fibrosis with thickened alveolar membranes and interstitial remodeling that lead to impaired gas diffusion and decreased lung compliance [58].

Finally, the coronavirus itself presumably possesses profibrotic characteristics. Nucleocapsid of SARS-CoV was documented to directly promote TGF-*β* pathways [58, 63]; thereby, it may be expected that SARS-CoV-2, owing to the significant similarities of their nucleocapsid protein (90%) [64], also endorses profibrotic signaling. Moreover, the other proposed mechanism for coronavirus-induced TGF-*β* upregulation appears to be indirect, *via* angiotensin II, knowing that coronaviruses downregulate angiotensin-converting enzyme (ACE) 2 and diminish angiotensin II clearance in the lungs [58].

Taken together, it is meaningful to expect post-COVID fibrosis development. Owing to their pathological characteristics, fibrotic changes are unlikely to be resolved, particularly in COVID-19 patients who were confronted with severe clinical forms and a longer duration of the disease or who potentially have an underlying lung disease [65]. Therefore, a biologic rationale exists that antifibrotic therapy may prove beneficial for COVID-19 patients, particularly if employed in a timely manner within the onset of the first symptoms of a cytokine storm [65]. Pathophysiologically speaking, if antifibrotic therapy is to be effective, it should equally and meticulously address anti-inflammatory and antifibrotic pathways while mitigating the fibrotic outcome. As evidenced, transforming growth factor *β* (TGF-*β*) signaling pathways, including both canonical (Smad-dependent) and noncanonical phosphatidylinositide 3 kinase, protein kinase B (PI3K/Akt), extracellular signal-regulated kinases 1 and 2 (ERK 1/2), and p38 mitogen-activated protein kinase (MAPK) are majorly attributed to fibroproliferation; therefore, a pharmacological targeting of these pathways should prove effective [15]. In this regard, renalase possesses putative traits that might prove valuable in inhibiting some of these mechanisms. Experimental data, so far, provide a theoretical basis to support the belief that exogenous renalase administration may prevent interstitial fibrosis of various organs [20–22, 29, 30, 34, 36–38].

## 3. Recognition of Renalase's Remote Roles

Renalase, a relatively “young” flavoprotein, was introduced into the scientific world in 2005, and was identified as the flavin adenine dinucleotide (FAD)-nicotinamide adenine dinucleotide (NADH) oxidase [66–70]. The discovery of renalase was based on premises about a molecule having a potential purpose to renal and cardiovascular health, while at the same time reflecting the function of the cardiorenal axis [66–68]. Renalase, derived from the kidneys, has been evidenced to be upregulated under any kind of stress condition, with the intention of systemic hemodynamic improvement [66–70], and cell survival [18–43, 69, 70]. Its remarkable expression in proximal tubules initially led to the conclusion that renalase circulating levels depend solely on factors associated with the kidneys, such as renal function and renal perfusion [70]. Additional studies, however, provided evidence that renalase tissue distribution is spread far beyond the kidneys, and that it is, to varying extents, expressed in the heart, liver, small intestine, skeletal muscles, endothelium, brain, and peripheral nerves, whereas the most recent research confirmed its presence in malignant tissue as well [68–77]. The most recent research implies a presumable role for renalase in placental development and function [41], owing to its placental expression throughout human gestation.

The human renalase gene is located on chromosome 10, and it contains 11 exons [42, 67]. There are two major isoforms of human renalase, known as renalase 1 and 2 [42], and two more isoforms (3 and 4), that lack oxidase function activity, due to their significantly shortened amine oxidase domains [18, 42, 68]. However, the existence of these variants substantially delays the recognition of renalase-dependent signalization, and mechanisms of action, which to date remain not fully elucidated. Renalase has been initially identified as a prominent contributor to the homeostasis of circulating catecholamines, following the discovery that their increased plasma concentration upregulates the synthesis, secretion, and activity of renalase [66, 67, 78]. However, this was proven for circulating renalase [78], while the specific roles of its, evidently present, intracellular forms remained vague [69]. Renalase's role in hemodynamic protection may be briefly explained by its circulation in the blood as prorenalase, waiting for an adequate signal (elevated blood pressure, catecholamine surge, or an organ injury) for its prompt turnover [66, 67, 69, 70, 78]. These stress signals also induce *de novo* renalase synthesis and secretion [78]. However, it is revealed that renalase metabolizes catecholamines, and catecholamine-like substances, *via* a superoxide (O2^–^)-dependent mechanism, with nicotinamide adenine dinucleotide phosphate (NAD(P)H) as a co-factor [43, 68, 78]. More recent research documents that renalase converts endogenous dihydro forms of *β*-NAD(P)H to metabolically available, noninhibitory *β*-NAD(P)H form [43]. Outstanding progress in research regarding the pathophysiology of renalase evidenced that this protein may act in, at least two, independent ways: as an enzyme, as briefly described, and as a cytokine, independently of its catalytic properties. Renalase gene expression is regulated by a range of transcription factors, including signal transducer and activator of transcription (STAT) 3, nuclear factor (NF)-*κβ*, hypoxia-inducible factor (HIF)-1*α*, tumor necrosis factor (TNF)-*α*, Kruppel family member zinc binding protein 8 (ZBP89), and specificity protein (Sp)1, several of which are related to inflammatory response [43]. Upon upregulation, renalase exerts its multifaceted behavior through the activation of its receptor, identified to be the plasma membrane Ca^2+^-ATPase 4b (PMCA4b) [25, 43, 70], and this signaling is linked to the stimulation of multiple downstream triggers, such as STAT3, NF-*κβ*, extracellular signal-regulated kinase 1 and 2 (ERK1/2), p38, cyclic adenosine monophosphate (cAMP), phosphatidylinositol 3-kinase/protein kinase B (PI3K/Akt), and Ca^2+^. The unique conclusion of all research is that renalase, after triggering its receptor (PMCA4b), sets in motion several molecular pathways, which aim to modulate “renalase-dependent” signaling, in order to exert pleiotropic, presumably context- and time-dependent actions, towards cytoprotection, and will be comprehensively discussed throughout this review. Finally, notable tissue distribution, substantial circulating forms, and remote mechanisms of action indicate systemic function for renalase, supporting the hypothesis of its important role in an organ protection following injury, and presumably in COVID-19.

## 4. Renalase Possible Cross-Linking with COVID-19-Related Cytokine Storm Components

The basic pathophysiology of COVID-19 may be summarized as follows: SARS-CoV-2, after entering the respiratory cells throughout the association of its protein S and a angiotensin-converting enzyme (ACE)2, as a receptor, expressed in different tissues (lungs, kidney, heart, and intestines) [9], triggers an immune response, particularly the toll-like receptor 7 on macrophages, resulting in the activation of several transcriptional factors, NF-*κβ*, provoking a surge of proinflammatory cytokines (IL-1*β*, IL-6, IL-12, IL-18, IL-33, TNF-*α*, and TGF-*β*) and interferon regulatory factors (IRFs) that induce interferon expression [7–14]. At the same time, anti-inflammatory cytokines, IL-4 and IL-10, in particular, are also significantly elevated, aiming to suppress the inflammation [8]. These systems synergistically, if uncontrolled and impaired, maintain disproportionate and excessive systemic inflammatory responses [7–14], resulting in massive neutrophil and macrophage lung infiltration [8–10, 12], followed by a remarkable production of reactive oxygen species (ROS), and subsequent cell apoptosis and necrosis [17]. Taken together, redundant innate immunity, and its related consequences, particularly oxidative stress, may be pivotal players in the pathophysiology of lung injury induced by respiratory viruses [12–14, 17], including SARS-CoV-2.

In line with these findings, a growing body of research evidenced that renalase confers impressive cytoprotective traits, in a manner far more complex than simply being a catalytic enzyme, offering a possibility that its supplementation during the course of various diseases, and hypothetically COVID-19, may provide some beneficial, anti-inflammatory, and antioxidant effects.

Notably, the discovery that the promoter region of the renalase gene contains four plausible targets for hypoxia-inducible factor (HIF)-1*α*, demonstrating a significant and positive correlation with HIF-1*α* expression in the setting of ischemic myocardial damage [79], led to the hypothesis that renalase may be a significant pathogenic factor involved in the pathogenesis of hypoxia [23, 24, 28, 35]. HIF-1*α*/renalase axis presumably functions with the purpose of compensation, and/or adaptation to a low oxygen environment, supporting the possible link of renalase and COVID-19-related ARDS. However, HIF-1*α* represents a ubiquitous transcriptional factor that is a key regulator of cellular actions as a response to hypoxia, whose activation results in the transcription of different genes, with renalase being among them. The same research reported that in the experimental model of an ischemic cardiac injury, premedication with recombinant renalase resulted in diminished myocardial injury (e.g., the size of the infarction area) and in significantly improved heart function. An appealing theory of renalase's pathophysiology was offered, presuming that its antihypoxic traits rely on renalase's capability for energy metabolism regulation during hypoxia, particularly in modulation of nicotinamide adenine dinucleotide (NAD^+^) and ATP levels in cells [79]. Similar findings were evaluated and confirmed, *in vitro* and *in vivo*, in a similar hypoxic milieu, of an experimental model of ischemic kidney injury [80]. Particularly, renalase was once again confirmed to be subjected to HIF-1*α* regulation at transcriptional level, presumably for reasons of delayed cytoprotection in the hypoxia.

These observations may be of a particular interest for this current pandemic, knowing that hypoxia represents initial pathophysiological phenomenon during COVID-19, and is attributed to all clinical forms of the disease [81]. However, some authors offered alternative hypotheses of COVID-19 pathogenesis, presuming that HIF-1*α* activation occurs early during the course of the disease, initiating a few meaningful pathways [81–83]. The first suggests that activation of HIF-1*α* signalization results in the activation of innate immune cells, leading to increased cytokine production, and tissue macrophage accumulation, while the second mechanism proposes involvement of endothelial cells, followed by increased expression of vascular endothelial growth factor and integrins, leading to increased vascular permeability and subsequent neutrophils accumulation [81–83]. Taken together, HIF-1*α* is greatly involved in the cytokine storm drive, and it is reasonable to believe that, in a similar manner, it induces renalase transcription, aiming to compensate for hypoxia, and maybe even to control or mediate possible detrimental effects of proinflammatory stimuli, and leukocytic lung accumulation, induced by HIF-1*α*.

Besides HIF-1*α*, the other crucial transcriptional factor for renalase gene expression was discovered to be a nuclear factor (NF)-*κβ*, yet again confirming that renalase may have a prominent role in inflammatory, and oxidative mechanisms modulation [31, 32, 73], beyond only being an anti-ischemic cytokine. NF-*κβ* functions as a powerful regulator of inflammatory and antiapoptotic gene expression, and is commonly activated upon proinflammatory signals, such as cytokines, antigens, and bacterial products, and hypoxia, in order to inhibit apoptosis, and to promote cell survival [84]. At the same time, NF-*κβ* evokes the transcription of proinflammatory cytokines, adhesion molecules, and enzymes involved in inflammation that, as evidenced, may be renalase [31, 32, 73]. For instance, in stress conditions, epinephrine, as demonstrated, triggers remarkably expressed *α*-adrenergic receptors in the proximal tubular epithelial cells, resulting in the NF-*κβ* activation and subsequent renalase secretion [31]. Similarly, proinflammatory IL-6 secretion is managed *via* the same *α*1-adrenergic receptors' stimulation, through p38 MAPK and NF-*κβ* pathways [85], so it may be speculated that renalase and IL-6 share similar signaling transduction [18, 69, 70, 86]. Moreover, the cytokine storm in COVID-19 is characterized by the ability of NF-*κβ*, and signal transducer and activator of transcription 3 (STAT3), to amplify the actions of IL-6, in order to further induce numerous proinflammatory cytokines, including IL-6 and monocyte chemoattractant protein (MCP)-1 [8–11]. It may be hypothesized that the same pathways (NF-*κβ* and STAT3) induce renalase transcription, most likely aiming to control, and even suppress inflammation, as STAT3 was also recently evidenced as a transcriptional factor for renalase [86]. The theory of renalase anti-inflammatory power may be compelling for COVID-19 outcomes, knowing that during the cytokine storm, the anti-inflammatory cytokines levels (IL-4 and IL-10) are increased [8], but lacking full anti-inflammatory capacity. The theory of possible renalase/IL-6 shared pathways may be interpreted in a similar manner as the aforementioned HIF-1*α*/renalase axis, in the context of renalase being a mediator of inflammation. Presumably, in the conditions of pathologic hyperinflammation, as presented in the cytokine storm, HIF-1*α*, IL-6, and renalase are all supposed to participate significantly, but have never been linked before in such a context. The recognition of HIF-1*α* and IL-6, as being important pathogenetic factors in COVID-19, gives renalase a potential, yet unrecognized, role in COVID-19. Finally, pharmacological suppression of cytokines during hyperinflammatory response may increase the risk of secondary infection [11], whereas the inhibition of particular IL-6 did not, so far, confirm a survival benefit in SARS-CoV-2-infected individuals [87, 88]. Moreover, the inhibition of IL-6 has been confirmed to exert both profibrotic or antifibrotic traits, regarding the phase of the acute inflammation in which its suppression was achieved [65]. In this context, it would be intriguing to research whether renalase may modulate IL-6 release, the mechanisms, and extent to which it may act as a modulator of severe SARS-CoV-2 infection.

In addition to the aforesaid, TNF-*α*, a cytokine greatly involved in the modulation of inflammation, infection, apoptosis, and acute organ injury is also evidenced to be responsible for renalase upregulation [32]. Indeed, stress signaling, such as hypoxia, leads to a release of TNF-*α* from the injured tissues into the circulation, resulting in activation of NF-*κβ* and subsequent renalase secretion. In line with these factors, pharmacological inhibition of both, TNF-*α* (Humira) and NF-*κβ* (pyrrolidine dithiocarbamate, PDTC), signaling pathways significantly hinders renalase expression [32]. Moreover, it is widely recognized that TNF-*α* may cause adverse effects through its inadequate and inappropriate actions, as being one of the most important cytokines during the cytokine storm, as described above. However, this particular research [32] confirmed that TNF-*α*/NF-*κβ*/renalase axis exhibits favorable outcomes, summarized as reduced cell cytotoxicity (validated as reduced LDH levels), and apoptosis (measured as decreased caspase-3 activity), a phenomenon that may be of a great advantage in COVID-19. It was recently confirmed that renalase is upregulated following stress signals, such as experimental fasting, aiming to protect intestinal cells from injury. Increased small intestinal renalase expression was observed following NF-*κβ* p65 upregulation [73], further supporting a hypothesis of renalase as a powerful protective protein. Discussing the context of acute inflammation and renalase's hypothetical role within, it should also be mentioned that increased plasma catecholamines strongly contribute to the progression of the pathological inflammation, and multiorgan dysfunction in the cytokine storm, by activating *α*-adrenergic receptors expressed on leukocytes [89]. Knowing that renalase acutely metabolizes catecholamines, once again, signposts its role in the pathophysiology of the cytokine storm.

**Figure 1:**
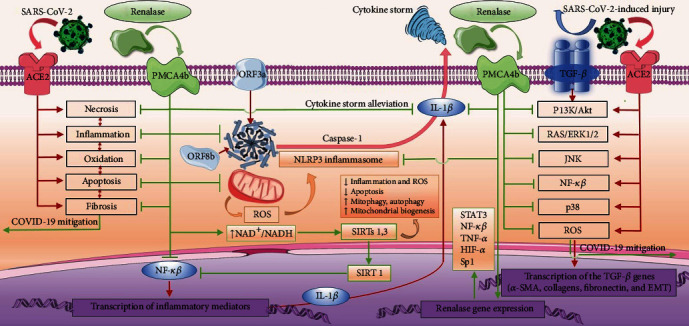
A schematic view showing the anticipated cyto-protective properties of renalase, indicated as a state of antinecrosis, anti-inflammation, antioxidation, antiapoptosis, and antifibrosis, and SARS-CoV-2-induced activation of the TGF-*β* receptor, followed by the upregulation of several intracellular signaling cascades, together with renalase's action points that may prove beneficial for COVID-19 mitigation and cytokine storm alleviation. Evidence has shown that renalase lessens necrosis; diminishes total neutrophil and macrophage accumulation, while promoting the anti-inflammatory (M2) macrophage phenotype (M1/M2 polarization), thus blocking the inflammasome activation, and IL-*β* production; and downregulates the expressions of MCP-1, MIP-2, and NADPH oxidase components (gp91^phox^, p47^phox^, and p67^phox^), while abolishing MDA and promoting SOD expression. Besides, renalase provides antiapoptosis, specifically by suppressing cyto C liberation, and Bax expression, while elevating Bcl-2 expression, and antifibrosis, by means of epithelial to mesenchymal transition silencing, decreasing the expression of *α*-SMA, collagens (I, III), TIMP-1, and TGF-*β*1, while restoring E-cadherin and increasing MMP-1 expression. At the same time, renalase upregulates the actions of sirtuins 1 and 3 (NAD^+^-dependent type III deacetylases), by increasing their co-substrate NAD^+^ concentrations. In that manner, renalase presumably provides an indirect role in SIRT1 protection, including decreasing NLRP3 inflammasome activation, oxidative stress, apoptosis, inflammation, and fibrosis reduction, as well as increasing the processes of autophagy and mitophagy, thereby preserving mitochondrial biogenesis and dynamics. Furthermore, renalase promotes SIRT3 activity, thus likely upgrading its protective effects, such as antiapoptosis, antihypertrophy, and antifibrosis, as well as suppressing mitochondrial ROS production, while silencing the inflammasome activation, impeding ERK1/2 and PI3K/Akt signalization, and increasing autophagy, hence, further promoting mitochondrial biogenesis and dynamics. In addition, the activation of TGF-*β* (by SARS-CoV-2) triggers the noncanonical network (PI3K/Akt, ERK1/2, JNK, p38, NF-*κβ*, and ROS), providing a cascade of fibroproliferative actions, and differentiation of fibroblasts, which may be, at various sites, modulated by renalase. Presumably, regulation of the TGF-*β* noncanonical signaling by renalase occurs within a context-dependent manner (activation or inhibition), to hindering epithelial to mesenchymal transition, profibrotic gene expression (*α*-SMA, collagen I and III, TIMP-1, and fibronectin), and restores E-cadherin, leading to a decreased extracellular matrix deposition and increased MMP-1 expression. Renalase gene expression is regulated by transcription factors, such as STAT3, NF-*κβ*, HIF-1*α*, Sp1, and TNF-*α*, while its signaling capacities, through its receptor PMCA4b, are linked to mediation of downstream signals, including STAT3, NF-*κβ*, ERK1/2, p38, PI3K/Akt, cAMP, and Ca^2+^. ORF3a and ORF8b are proteins encoded by SARS-CoV-2 promoting the assembly of NLRP3 inflammasome, whose activation and IL-1*β* production may be blocked upon the administration of renalase, which may be a step to improving the treatment success rate of patients with severe COVID-19. SARS-CoV-2: severe acute respiratory syndrome coronavirus 2; TGF-*β*: transforming growth factor-*β*; PMCA4b: plasma membrane calcium-ATPase 4b; MCP-1: *monocyte chemoattractant protein-1*; *MIP-2*: macrophage inflammatory protein-2; NADPH: nicotinamide adenine dinucleotide phosphate; MDA: malondialdehyde; SOD: superoxide dismutase; TIMP-1: tissue inhibitor of metalloproteinase-1; MMP-1: *matrix metalloproteinase-1*; *α*-SMA: *α*-smooth muscle actin; NAD: *nicotinamide adenine dinucleotide*; ORF3a: open-reading frame 3a; ORF8b: open-reading frame 8b; PI3K/Akt: phosphatidylinositol 3-kinase/protein kinase B; ERK1/2: extracellular regulated protein kinases 1/2; JNK: c-Jun N-terminal kinases; NF-*κβ*: nuclear factor *κβ*; ROS: reactive oxygen species; Sp1: specificity protein 1; EMT: epithelial to mesenchymal transition.

These understandings may be finally underlined with the most recent results of the pioneer studies of renalase's role in the SARS-CoV-2-related pandemic and interpreted through the context of the aforementioned postulations [90–92]. It was, however, evidenced that renalase plasma levels measured in COVID-19 patients were significantly decreased compared to that of the control group, and demonstrated a negative correlation with inflammatory cytokines plasma levels, namely, IL-1*β*, IL-6, and TNF-*α* [90]. At the same time, decreased plasma renalase was associated with more severe clinical manifestations of COVID-19, and worse survival rates (HR=4.54), strongly supporting a theory of renalase being a useful biomarker additive for the identification of the most severe COVID-19 patients [90]. Its additional value may facilitate the identification of patients who are more likely to progress to acute lung injury, and COVID-related ARDS, and so in a timely manner, providing them with the proper therapy. Renalase was, however, suggested to be a factor in the survival of COVID-19, whose plasma levels most probably rise in order to counteract inflammatory marker levels, supporting the evidence of renalase's anti-inflammatory traits. The additional study [91] further supports the hypothesis that sufficient renalase plasma levels are important for biological integrity and injury responses. Patients presented with increased renalase/decreased IL-6 (established marker of mortality in COVID-19) plasma levels had the best survival compared to other evaluated groups, while decreased renalase concentration (“blunted response”) experienced worse outcomes, as defined by higher mortality, greater hypoxia, a longer length of stay, higher intensive care unit admission, use of vasopressors, and cardiopulmonary resuscitation rate [91]. Moreover, every 1000 ng/ml increase in renalase levels decreased the risk of death or intubation by 5% (HR=0.95) and increased survival alone by 6% (HR=0.95), after adjustments for socio-demographics, initial disease severity, comorbidities, and inflammation. Finally, renalase was independently associated with reduced intubation and mortality in hospitalized COVID-19 patients. The most recent research regarding renalase presumable role in the pathophysiology of COVID-19 [92] revealed that its levels significantly increased from the beginning of observation (day 7 to 14), reaching its highest levels on day 28, presuming that renalase upregulation is most likely related to the activation of the immune system, rather than kidney function [92]. A summary of current research regarding the assessment of renalase in COVID-19 hospitalized patients is presented in Table 1, and the proposed mechanisms of renalase in COVID-19 mitigation are depicted in Figure 1.

Taken together, regarding the pathophysiology of COVID-19, decreased renalase plasma levels may be a relevant pathogenic factor (or a biomarker) resulting in (or predicting) a poor outcome, or may be relevant to guide patients' management, and therapeutic approach. These observations warrant much-needed research in patients with severe COVID-19, in analyzing whether renalase administration may probe the roles of proinflammatory cytokines, thus silencing inflammatory cascade in the severe COVID-19, to effectively reduce acute disease severity, as demonstrated in preclinical models.

## 5. Renalase's Pathways and their Association with COVID-19's Signaling Cascade Activity

Renalase-dependent cell signaling may also be debated in the context of COVID-19, since the triggering of renalase receptor, PMCA4b [18, 25], *in vitro*, results in a rapid increase of phosphorylated ERK1/2, p38, and PI3K/Akt, while downregulating c-Jun N-terminal kinases (JNK) [18, 25, 69, 70]. Activation of ERK1/2 and PI3K/Akt pathways is particularly beneficial during a hypoxic injury, as renalase may enhance cell survival by inhibiting the signals that favor the death of the cells, such as MAPKs and PI3K/Akt [18, 76, 93]. These findings may be particularly captivating for the possible clinical employment of renalase during the course of COVID-19, considering that by shifting the balance between ERK1/2 and JNK activation towards increased ERK1/2 and PI3K/Akt activation, the cells will presumably overcome and survive oxidative damage [93]. Accordingly, p38, PI3K/Akt, and ERK1/2 pathways, which are evidenced to be the most strongly regulated kinases [6], are remarkably involved in cell signaling during the COVID-19-related cytokine storm [6, 11]. It is broadly acknowledged that these signaling pathways are responsible for cellular responses, such as survival or mortality [94]. Moreover, in SARS-CoV-2 infection, it was evidenced that the particular p38 MAPK pathway mediates pathogenic infection cell response and potentially harmful proinflammatory cytokine stimulation and very likely results in cell cycle arrest [6]. Moreover, as stated, some viral infections and SARS-CoV-2, respectively, induce a p38 MAPK signaling condition, resulting in an uncontrolled positive feedback regulation and hyperinflammation associated with severe disease [6]. However, according to the analysis of the kinase activity of SARS-CoV-2 phosphorylation profiles, an upregulation of some components of the p38 signaling has been reported in COVID-19, indicating that SARS-CoV-2 infection promotes p38 MAPK activity [6]. In COVID-19, notably, blocking of this signaling might decrease proinflammatory cytokine production, and lessen viral replication, through an as yet unidentified mechanism, indicating that suppression of p38 MAPK may favor the outcome in SARS-CoV-2 related infection [6]. As evidenced, transcription factors regulated by p38 are remarkably effective following the infection [95], providing an indirect, but plausible role for renalase in SARS-CoV-2-related disease. Owing to the multifaceted nature of renalase, its substantial interference (activation or suppression) with the p38 MAPK pathway is presumably within a context- and time-dependent manner. The MAPK activation *via* renalase supplementation has been proven to be protective [18, 25], whereas its inhibition, which may be distinctly important during SARS-CoV-2 related infection [6], has also been confirmed [20, 33, 34, 36], and this type of response and its plausible utility in COVID-19 will be thoroughly discussed in the final section of the review. A schematic view showing signaling pathways of renalase that may be associated with COVID-19 signaling activity is presented in Figure 1.

## 6. Renalase's Antioxidative, Anti-inflammatory, and Antiapoptotic Roles in the Context of COVID-19

Increasing evidence supports the hypothesis that exacerbated reactive oxygen species (ROS) production acting synergistically with the deterioration of antioxidant activity represents a notable mechanism in the pathophysiology of SARS-CoV-2-related disease, as well as in the progression of the respiratory failure, causing lung damage, and eventually death [17, 96, 97]. Reactive oxygen species during COVID-19 may originate from various sources. The first source may be activated neutrophils and macrophages, being a part of their virucidal and bactericidal activity, aiming to destroy the phagosome trapped pathogens [9]. Secondly, by virus generation, in order to invade host cells or following the interaction between the human metabolism and the pathogen, and finally, through the evidenced mitochondrial disruption [96]. Viruses significantly interfere with mitochondrial homeostasis in order to avoid the host's antiviral response and to protect the process of their own replication [96, 97]. For instance, an identified protein, assigned as open reading frame (ORF)10, encoded by SARS-CoV-2, suppresses the antiviral innate immune response by degrading mitochondrial antiviral signaling protein (MAVS) through the ORF10-induced autophagy pathway, to help evade the host's innate immunity, resulting in significant ROS production, and formation of a proinflammatory and apoptotic environment [98]. SARS-CoV-2, in particular, after binding for its ACE2 receptors, leads to increased production of ROS followed by cell damage, automatically initiating a vicious oxidative stress round, contributing to a worse prognosis of COVID-19 [99]. Additionally, ROS may oxidize the cysteine residues of ACE2 receptors and the receptor-binding domain of the SARS-CoV-2 S protein [100], keeping them in oxidized forms. This may, however, favor the affinity of ACE2 for SARS-CoV-2 S protein [99, 101], once again generating an unfavorable COVID-19 outcome [99, 101]. Coupled with that, ROS clearly activate the transcription of NF-*κβ* and HIF-1*α*, which are evidenced transcriptional factors for renalase expression [42, 70, 86], providing a plausible link between ROS generation, oxidative environment-induced redox-sensitive transcriptional factors, and renalase. Moreover, ROS along with proinflammatory cytokines also accelerate a profibrotic lung response, regardless of the fibrogenesis pathway activated [15, 16], clarifying the role of ROS in an inflammation-driven lung fibrosis.

Nevertheless, studies in rats, *in vivo*, demonstrated that, beside catecholamine infusion that promptly activates circulating forms of renalase [78], renalase elevation was presumed to be induced by another, undiscovered trigger. The recognition that oxidative stress induces renalase expression in the cell culture [68], gave support to the remarkable research on renalase pathophysiology, establishing previously unrecognized mechanisms of its antioxidation pathways. The most recent research demonstrated, *in vivo*, renalase antioxidant properties in the small intestine [73]. Nonetheless, the research evidenced that fasting-induced oxidative stress significantly increases renalase intestinal expression, induced by NF-*κβ* p65 activation, whereas at the same time, renalase evidenced positive correlation with the degree of oxidative stress, namely, with increased markers of lipid peroxidation and carbonylated proteins. The upregulation of p65 promotes antioxidant gene expression in line with renalase for maintenance of antioxidative activity. A very appealing concept was established by observing that renalase was more uniformly expressed in the ileum compared to the jejunum. Nevertheless, the ileum represents a part of intestine that is specifically included in immunity, rich in intestinal bacteria that likely make the response to oxidative stress sensitive, underpinning renalase as the final defense tool for oxidative stress cell protection, resembling the function of glutathione peroxidase [73]. Similarly, renalase's antioxidative ability was validated in the liver tissue, whereas few additional hypotheses of its pathways were proposed [72]. Nevertheless, a strong and positive association between hepatic cells' renalase expression and hydrogen peroxide, H_2_O_2_ (a derivate of superoxide anion, O_2_^−^), concentration was established, including the incubation time period in the culture of the hepatocellular carcinoma cell line subjected to oxidative damage. Such an outcome may be attributed to renalase's powerful and prompt responsiveness to a burst of ROS, whereas renalase feedback was confirmed to be dependent upon the intensity and duration of the oxidative injury [72]. Nonetheless, H_2_O_2_ is considered to be a stable and milder form of reactive oxygen species, but may be easily converted to hydroxyl radicals, more reactive and more toxic forms of ROS [17]. Besides, in the settings of *in vitro* antioxidants preincubation superoxide dismutase (SOD) and catalase (CAT), the same antioxidants' intraperitoneal preinjection *in vivo* renalase cell expression was significantly mitigated, implying that renalase may be successfully abolished by antioxidative therapy. Moreover, serum levels of the liver enzymes, indicating necrosis of the hepatocytes (AST, ALT, ALP, LDH, and *γ*-GT), and serum renalase positively correlated, whereas all concentrations significantly decreased when antioxidant therapy (SOD and CAD) was administered. Renalase, owing to its prompt reaction to oxidative stress, warrants primacy in becoming a marker for the severity of oxidative tissue injury and for assessment of the effects of applied antioxidant therapy [72]. Related research tested the hypothesis whether renalase may suppress reactive oxygen species generation by improving mitochondrial function and, if so, to identify the precise pathophysiological pathways [19], considering this to be a commonly used protection strategy for renalase. Understanding that mitochondria represent a substantial origin of ROS, and at the same time being the very first target of their detrimental actions, led to the theory that renalase antioxidative actions may improve the function of mitochondria, *in vivo*, and, *in vitro*, in liver ischemic injury. Renalase was validated as directly mitigating cellular and mitochondrial ROS production, lessened malondialdehyde (MDA) formation, and boosted SOD activity and glutathione (GSH) levels [19]. By improving mitochondrial function, renalase, at the same time, indirectly prevents oxidative bursts and subsequent apoptosis, recognizing that damaged mitochondria release proapoptotic factors [19]. Indeed, the study confirmed that renalase administration was followed by preserved cellular ATP content, mtDNA copy numbers, suppressed cell apoptosis (by impediment of cyto C liberation, and Bax expression, and elevating Bcl-2 expression), alleviated mitochondrial fission, and finally reduced ROS generation [19]. A notable correlation was made that renalase most likely rescues the mitochondrial phenotype by attenuating its fission, and reduced ROS-generated apoptosis, *via* activation of sirtuin (SIRT)1. For better clarification of these premises, SIRT1 is known to be the NAD^+^-dependent type III deacetylase that is notably involved in cell survival and metabolic homeostasis [102, 103]. Indeed, in those settings associated with ischemic injury, SIRT1 deactylates NF-*κβ*, among other downstream targets, for the management of inflammation, oxidative stress, and apoptosis [102, 103]. The hypothesis of a relationship between renalase and sirtuins and its possible employment in COVID-19 will be discussed more specifically later.

One of the first experimental models while testing the hypothesis of renalase's antioxidant properties postulated a few theories [24] that were broadly assessed afterwards. It was, however, confirmed that a prompt increase in plasma renalase levels represents the first response to a cardiac ischemic injury and that recombinant renalase administration rescued cardiac phenotype efficiently. Furthermore, the same study revealed that knockout animals exhibited very severe cardiac necrosis, shown to be three times more severe compared to controls, after being subjected to ischemic damage, with a complete recovery after renalase utilization. The hypothesis of renalase's protective pathway was postulated for the first time, by its effects of extracellular NAD^+^ levels, and cellular redox metabolism [24]. Nevertheless, the lack of a renalase gene in knockout animals was associated with significant decline in plasma NADH oxidase activity and in the NAD^+^/NADH ratio within the cells. At the same time, it was discovered that renalase functions as an oxidase/anomerase, utilizing molecular oxygen for conversion of *α*-NAD(p)H to *β*-NAD+ [104], which allowed a clearer understanding of renalase's protective response [18, 24]. More precisely, the knockout animals experienced a decreased ratio of oxidized (NAD^+^) to reduced (NADH) nicotinamide adenine dinucleotide in the damaged tissue [24]. Many studies have confirmed the relevance of NAD^+^ in energy metabolism and electron transfer reactions [24, 102, 103, 105, 106], whereas its elevated levels of NAD^+^ may upgrade SIRT1 activity [24, 102, 103, 105]. It deserves mentioning that NAD^+^ possess essential function in the positive regulation of SIRT1 [102, 103, 105]. According to the report, circulating renalase functions as plasma NADH oxidase, so it may be concluded that this protein is significantly responsible for sustaining extracellular levels of NAD^+^ [19, 24]. Moreover, in leukocytes, NAD^+^ functions as a receptor in adhesion and signaling pathways, whereas both NAD^+^ and NADH have relevant protective roles in processes, such as oxidative stress and mitochondrial dysregulation, and improve the function of NAD^+^-dependent sirtuins [105].

In line with this, pronounced biological actions of NAD^+^ are enhanced through its endogenous synthesis [105] and purportedly *via* renalase enhanced secretion and/or administration. In the opposite setting, decreased NAD^+^/NADH ratio favors organ injury in a low oxygen environment, implying that the same mechanism of injury may be, at least in part, responsible for hypoxia-induced lung damage during COVID-19. Accordingly, a very appealing discovery of unbalanced p53/SIRT1 axis and its impact to lymphocyte homeostasis (cell survival, B cell signaling, and antibody production) in severe acute respiratory syndrome SARS-CoV-2-related infection was recently offered [107]. SIRT1 expression was significantly decreased in COVID-19 patients, and at the same time negatively correlated with p53 and the concentration of proinflammatory cytokines [107], offering an indirect role for renalase employment as a regulator of NAD^+^ [19, 24] for SIRT1 upregulation in cases of severe COVID-19 [107].

Similar renalase antioxidative traits were evidenced in the related research of renal ischemic injury [23], likely obtaining more extensive cytoprotection, considering that ischemic renal injury represents a systemic condition, followed by an intrarenal accumulation of proinflammatory leukocytes, systemic inflammation with multiple systems involved, including the lungs, liver, and intestine [108]. These extrarenal complications are considered to be life-threatening and responsible for high mortality [23]. The study clearly demonstrated that renalase administration significantly impeded renal accumulation of neutrophils and macrophages, and relieved both necrosis and apoptosis of renal tubular cells, a similar pattern presented in the lungs of COVID-19 patients. Accordingly, renalase was evidenced to exhibit kidney protection addressing the most powerful pathways of cell trauma: oxidative stress and inflammation leading to cell death, necrosis, and apoptosis. When administered before, and in a short period of time (30 minutes) after ischemic injury, recombinant renalase significantly mitigated ischemic lesion in the knockout animals, addressing those deleterious pathways. In line with that, the lack of renalase gene in knockout animals resulted in increased levels of TNF-*α*, monocyte chemoattractive protein (MCP)-1, and macrophage inflammatory protein (MIP)-2, all markers of systemic inflammation, suggesting that renalase most likely rises to counteract proinflammatory cascade, therefore exerting anti-inflammatory effects [23]. Renalase overall cytoprotection has, once again, been validated *in vitro* and *in vivo* after toxic kidney injury [27]. In addition, a previously postulated theory that renalase preconditioning may provide several favorable effects has been further confirmed. In addition, the degree of oxidative stress was reduced, demonstrated by reduced malondialdehyde (MDA) levels and increased superoxide dismutase (SOD) activity and diminished renal inflammation, exhibited by decreased renal TNF-*α* levels, and MCP-1 expression, as well as reduced macrophages infiltration. Additionally, kidney function ameliorated, validated by restored serum concentrations of blood urea nitrogen and creatinine, whereas pathohistological changes (necrosis, foamy degeneration, and tubular detachment) were rescued. The same observations were confirmed *in vitro*, as renalase preconditioning decreased ROS expression and caspase-3 levels in the cell culture, once again proving that renalase exerts antioxidation, anti-inflammation, and antiapoptosis, and that may be valuable treatment in conditions related to this deleterious phenomenon, such as COVID-19. Additionally, renalase knockout animals had a significant elevation of cathecholamine plasma levels [23] that may also be debated within the context of renalase anti-inflammatory action. In particular, catecholamines are proven to be involved in the overall inflammatory response, whereas stimulation of their receptors, *α*1 and *α*2, expressed abundantly on numerous inflammatory cells [89, 109], contributes to an organ injury. Indeed, *α*1 receptor stimulation results in increased proinflammatory cytokine production in circulating monocytes and macrophages [89, 109]; therefore, it seems feasible that renalase “blockage” of adrenergic receptors to some degree provides organ safety [23]. Moreover, renalase supplementation may be an additional step in prevention of circulating catecholamines oxidation, since they are prone to autoxidation and subsequent ROS production, as already demonstrated in the pathogenesis of heart diseases [33], finally confirming that renalase's robust antioxidant capacity appears to be multifaceted.

Besides the reactive oxygen species formation, apoptosis was also confirmed to be one of the mechanisms for host invasion by SARS-CoV-2. However, SARS-CoV-2-encoded accessory protein, designated as ORF3a, was demonstrated to induce apoptosis, measured as activated caspase-3, a marker of caspase-dependent apoptosis [110, 111]. Coronaviruses-related apoptosis was however demonstrated to be induced by various mechanisms, whereas caspase-dependent apoptosis was described as an essential for the replication of the virus [17, 99, 111, 112]. If we are to introduce a beneficial therapeutic agent during COVID-19, the first phenomenon to address should be the unregulated release of reactive oxygen species, inflammation and apoptosis. Coupled with that, renalase knockout mice experienced a higher degree of apoptosis, significant macrophage infiltration, and more severe tubular necrosis, while at the same time, exogenous renalase administration prevented caspase-3 effector activation and increased antiapoptotic Bcl-2 expression [18]. The related research reported similar results that renalase inhibited cisplatin-induced upregulation of cleaved caspase-3 expression and downregulation of Bcl-2 expression [30]. Identified as a pleiotropic molecule, renalase appears to promote cytoprotection *via* various mechanisms, with antiapoptotic mediation being significantly expressed. As previously mentioned, renalase's modulation of apoptosis occurs through the augmentation of Bcl-2 expression, and hindering of caspase activation [18, 30, 32], an observation determinated in an experimental model of acute kidney injury, but may also be relevant within the cytokine storm. In addition, during toxic kidney injury progression, the degree of tubular apoptosis was significantly reduced after renalase administration, evidenced by reduced caspase-3 activity [27]. Finally, renalase deficiency, *in vitro*, promoted myocardial cells apoptosis and necrosis during an ischemic myocardial injury, whereas its administration significantly minimized the consequences of ischemia, including cardiomyocytes apoptosis [28]. A review of discussed effects of renalase based upon current data is comprehensively presented in Table 2, and proposed pathways are depicted in Figure 1. Taken together, renalase feasible ability to *in vitro* and *in vivo* reduces clinical perils by the reduction of ROS, inflammation, apoptosis, necrosis, and toxic catecholamine effects, which may be a brand new approach to battle COVID-19-induced cytokine storm and its detrimental consequences.

## 7. A possible Renalase/Macrophages Axis in the Context of COVID-19

As stated, the pathophysiological hallmark of the SARS-CoV-2-related infection represents extreme and rapid activation of the innate immune response [3, 7–14, 113], and subsequent detrimental increases in cytokine secretion, massive inflammatory cells lung accumulation, edema, and hypoxia, with subsequent acute lung injury, ARDS, and multiorgan failure (liver or kidney injury and/or myocardial ischemia) [113]. Moreover, this reaction in the lungs is greatly promoted by the alveolar macrophages, and monocyte-derived macrophage activated by various signals, released by apoptotic pneumocytes [8, 9, 15, 16]. The role of macrophages in the pathogenesis of the cytokine storm appears to be two-sided, regarding their additional stimuli of injury from the adaptive immune system, finally resulting in macrophage activation syndrome [8, 9, 113]. Regardless of the type of macrophage activation, the key step in the initiation of acute inflammation and subsequent cytokine storm is macrophage shift to the M1 subpopulation, generating overwhelming amounts of ROS and cytokines, with proinflammatory positive cascade feedback. In the context of SARS-CoV infection, reduction of the particular macrophage population (M1) alleviated lung inflammation drive, without influencing viral load [113]. Moreover, anti-inflammatory M2 macrophages were evidenced in the bronchioalveolar fluid of clinically improving SARS-CoV-2-infected patients [114]. Correspondingly, COVID-related cytokine storm presumably activated macrophages through HIF-1*α* upregulation [115], and undergoing inflammasome induction [116] mediates acute lung inflammatory response, progressing into ARDS. Therapeutic targeting of the particular inflammasome (NLRP3) pathway is currently in various stages of research [112] and is expected to show beneficial effects in patients with severe COVID-19. In addition, clear evidence exists that renalase suppresses inflammasome activity in activated macrophages [18, 26, 42] presumably controlling the release of cytotoxic mediators, namely, IL-1*β* and ROS, as evidenced [42]. This theory likely corresponds to the SARS-CoV-2-related inflammatory pathways, so it may be expected that renalase administration could weaken the inflammatory signaling that mediates acute lung injury and ARDS. Moreover, angiotensin II can induce inflammasome activation in the renal tubular cells [116], the very same site of renalase secretion [66–70], so this may provide another plausible link between inflammasome activation and subsequent renalase synthesis and secretion.

As previously stated, renalase knockout mice demonstrate a significantly increased expression of TNF-*α*, monocyte chemoattractive protein (MCP)-1, and macrophage inflammatory protein (MIP)-2, therefore a substantial renal [18, 20, 22, 23] and pancreatic macrophage accumulation, following various types of injury [26]. Accordingly, it was hypothesized that soluble renalase most probably targets various cell types, including inflammatory cells, principally macrophages [26]. Recognition that macrophages express the particular renalase-targeting PMCA4b receptors [117] further confirms the hypothesis that renalase may be essentially involved in the modulation of the acute inflammatory response, throughout the appropriate stimuli presented to macrophages, and their subsequent phenotype adjustment. Renalase activation of these particular receptors proved highly beneficial in rescuing pancreatic tissue during *in vivo* induced acute pancreatitis [26]. Based on this premise, it may also be concluded that the lack of circulating renalase can be correlated to more prominent tissue harm, since renalase knockout mice experience more severe macrophage infiltration [26]. Whether this PMCA4b activation initially endorses calcium efflux or synergistically affects renalase signaling pathways, namely, PI3K/Akt, ERK1/2, and cAMP levels [18, 69, 70], is yet to be clarified. In line with these reports, previous research confirmed that PMCA4b triggering, including renalase, enables PI3K/Akt-dependent mechanisms, with the intention of maintaining ATP levels, and presumably some other PI3K/Akt-independent pathways [26, 118]. These premises indicate that it is feasible to postulate that these receptors serve as a link between renalase and innate immunity, responsible for the cytokine storm [26]. In view of the fact that renalase most likely provides protection in the early phases of tissue injury, being linked with innate immunity [26], it may be concluded that all lesions subjected to innate inflammatory response, as well as lung injury related to cytokine storm in COVID-19, may benefit from timely renalase supplementation. Consistent with this, renalase was also highly associated with macrophages presenting with an anti-inflammatory M2 phenotype [26, 42], based on the presumption of its role in mediating and controlling macrophage phenotype shifting. Nevertheless, it was evident that if macrophages were depleted during the acute inflammatory phase, fibroproliferative response was alleviated, and myofibroblasts were decreased [16], and *vice versa*. A detailed summary of discussed research is presented in Table 3 and accordingly depicted in Figure 1.

Whether renalase may fine-tune the stimuli for macrophages to acquire a specific functional profile (pro- and antifibrotic) in the pulmonary tissue, in the same manner as demonstrated in the kidney, and pancreatic tissue, may be a captivating goal for more in-depth profiling to identify the proper renalase to macrophages signaling in the state of fibroproliferation and will be discussed more in the final section.

## 8. Renalase and Sirtuins Cross-Talk in the Context of COVID-19

A silent information regulator two proteins (sirtuins or SIRTs) are widely recognized as upregulators of various sets of proteins related to cells' prosurvival mechanisms [119–122], which provides a theoretical support for them being similarly involved in the survival pathways during COVID-19. Nonetheless, with regard to the novelty of the disease, very few studies of SIRTs' role in SARS-CoV-2-related disease have been reported or discussed so far [121, 122], whereas owing to their biological function, sirtuins' role in COVID-19 may be greatly anticipated. However, sufficient evidence already exists regarding their role within the pathophysiology of lung disorders. Indeed, various pharmacological upregulation of SIRT1 demonstrate significant effectiveness in lung protection, through the suppression of excessive inflammatory response, oxidative stress, apoptosis, and fibrosis. SIRTs-mediated mitigation of lung inflammation and cellular senescence is confirmed in settings such as obstructive pulmonary diseases [123], sepsis-induced acute lung injury [124], and ARDS [125]; idiopathic pulmonary fibrosis TGF-*β*1-mediated epithelial-mesenchymal transition and alleviation of its progression [126–128]; oxidative stress; and fibrosis in bleomycin-induced oxidative stress and pulmonary fibrosis [129], both the early (inflammatory) and late (fibrotic) stages of systemic sclerosis-related pulmonary fibrosis [130], indicating its potential employment as a therapeutic agent for pulmonary diseases with underlying inflammation.

The SIRTs family constitutes several proteins (1-7), whereas the best characterized sirtuin, (SIRT) 1, was documented to be a pleiotropic nuclear protein, promoting anti-inflammatory actions, *via* regulation of the effectiveness of NF-*κβ* signaling [131], and strongly regulating mitochondrial signaling [132]. Moreover, upregulation of SIRT1 may impede p53 pathways, resulting in antiapoptosis; it may also inhibit NF-k*β* of activated B-cells, providing anti-inflammation, which enable metabolic adjustment, survival, autophagy, and mitochondrial biogenesis; the actions required for cell survival [119–122], while, at the same time, downregulation of SIRT1, disturbs oxidative energy metabolism and stimulates NF-*κβ*-induced inflammatory responses [121]. The other member of the sirtuins family, SIRT3, for instance, is located within mitochondria, and its deacetylase activities significantly improve mitochondrial function [133, 134]. In addition, the activation of SIRT1/3 notably improves mitochondrial function [135], implying their evident role in the context of hypoxic and oxidative stress, mitochondrial dysfunction, and related apoptosis, inflammation, and immune function, being a putative pathway identified in the progression of COVID-19, with mitochondria being noted as relevant targets for treatment [96, 134]. Indeed, in the context of the current pandemic, SARS-CoV-2 virus has been proven to significantly alter mitochondrial dynamics on various levels, aiming for infection to progress [96], once again providing the support of the hypothesis which asserts sirtuins' involvement in COVID-19. Besides the aforementioned supervision of inflammatory processes, SIRT1, together with other sirtuins, represent an initial defense against both types (DNA and RNA) viruses, in general [121, 136]. Upregulation of SIRT1 exhibits direct inhibition of virus replication, followed by a decline in proinflammatory cytokine production, IL-1*β*, IL-6, and TNF-*α*, whereas downregulation of SIRT1 enhances replication of the viruses, causing an uncontrolled surge of proinflammatory cytokines [122], in a similar manner to that in the cytokine storm. Finally, SIRT1 activation remarkably inhibits oxidative stress [137, 138] in vascular endothelial cells [139] that are heavily involved in the pathogenesis of the cytokine storm in COVID-19, knowing that hyperinflammation-induced uncontrolled oxidative stress results in substantial endothelial cell damage [8–10, 12, 13], capillary leak and edema formation, and subsequent ARDS worsening [140]. Given the various SIRTs activities, in the context of COVID-19, their supervision of the host cell's metabolism may be an equally required element in the regulation of the interaction of the viruses and humans [121, 141].

In line with what we have previously discussed, an appealing hypothesis was postulated that renalase's ability to improve mitochondrial function, and at the same time provide antioxidative action, was due to its capacity to increase the activity of sirtuins 1 and 3, as documented up until now. However, being a family of NAD^+^-dependent protein deacetylases, sirtuins necessitate NAD^+^ for its activation [119, 120], as already stated. As conferred, renalase is verified to oxidize *α*-NADH converting it to *β*-NAD^+^ [104], so it may be concluded that renalase most likely activates SIRT1 *via* increasing levels of NAD^+^ [19, 24, 42], and *vice versa*, considering that the lack of renalase significantly downregulates NAD^+^ activity [24, 42]. It may be postulated that exogenous renalase administration may increase cellular NAD^+^ levels, and therefore, enhance SIRT1 activity, resulting in the deacetylation and modulation of the activity of SIRT1 downstream targets, particularly the peroxisome proliferator-activated receptor-*γ* coactivator 1*α*, the forkhead box O1, and O3 transcription factors [119, 120]. Moreover, as outlined previously, renalase administration rescues ischemic and oxidative stress injury phenotype *via* SIRT1 activation, evidently through the elevation of NAD^+^ levels [19]. This may be of a particular interest for the pathophysiology of COVID-19, especially since there is ample current evidence indicating that several viral pathogens, including SARS-CoV-2-related infections, significantly deplete cellular NAD^+^ levels [142]. Such unsteady framework, jointly with decreased NAD^+^ levels in older individuals, and in patients with comorbidities associated with severe COVID-19 symptoms, such as diabetes and cardiovascular disease, likely promotes the setting for detrimental cytokine storm [142]. Accordingly, several NAD^+^-dependent enzymes, including SIRT1, presumably inhibit the production of viral progeny [121, 142], supporting an intriguing theory of SIRTs as evolutionarily conserved viral restriction components [121, 143]. It deserves emphasis that some other NAD^+^-dependent enzymes, such as members of the poly-ADP-ribose polymerases (PARPs) families, exhibit antiviral potential, whereas SARS-CoV-2 proteins induce hyperactivation of PARPs, followed by cellular NAD^+^ depletion [144]. These observations, in line with renalase's capability to modulate the pool of NAD^+^, as evidenced, provide the proof of principle that renalase may be indirectly implicated in the modulation of sirtuins activities. In accordance with this feasibility, renalase was shown to protect against cisplatin-induced renal injury, by reducing mitochondrial fission and tubular epithelial cell apoptosis in a SIRT3-dependent manner, presumably *via* early MAPK signal activation [18, 25, 30].

Taken together, it is reasonable to believe that renalase's ability to fine-tune pleiotropic proteins, SIRT1 [19] and SIRT3 [30] regulation during the SARS-CoV-2-related disease, may be an additional factor aiming to inhibit viral reproduction and control inflammation and its consequences. Renalase, however, owing to its efficient sustaining of NAD^+^ levels may control SIRTs-mediated events during coronavirus 2 infection that, as stated, appear to be compelling participants in both inflammatory and fibrotic stages of pulmonary fibrosis [123–130]. The discovery of novel molecules, such as renalase, that interfere with SIRTs activities, possibly *via* regulation of NAD^+^ intercellular levels, providing advantage for sitruins' defense properties, may prove highly relevant against SARS-CoV-2 infection [121]. If verified, this hypothesis will provide large-scale benefits in the management of SARS-CoV-2-related disease. A schematic view of renalase and sirtuins interplay is depicted in Figure 1, and a comprehensive review based on current literature is summarized in Table 4.

## 9. A Potential Relationship of Renalase and Autophagy in in the Context of COVID-19

Taking into consideration the previously provided hypothesis regarding the potential renalase/sirtuin axis, another tempting, but as of now, only a theoretical approach may be offered that renalase, by upgrading sirtuin activity, likely modulates the process of autophagy. Beyond sirtuins, another possible link between renalase and autophagy arises from the fact that modulation of the PI3K/Akt signaling network, evidenced as a renalase downstream signal [18, 25, 42], as discussed, implies beneficial prospects as an autophagy-related approach [145]. This concept, which is admittedly solely based on current theoretical knowledge, may prove relevant in COVID-19 treatment, particularly considering that recent discoveries indicate an intricate and contributing interaction between coronaviruses (including SARS-CoV-2), and autophagy, thus proposing that the modulation of autophagy (either through the use of inducers or inhibitors) may be pernicious for viral replication, favoring a better patient prognosis in severe COVID-19 cases [145–152]. For instance, it has been documented that autophagy-inducing molecules may weaken the procreation of SARS-CoV-2 in primary human lung cells and intestinal organoids, indicating that these agents may be a feasible therapy option against COVID-19 [146]. Accordingly, pharmacological agents that induce autophagy generally antagonize the replication of coronaviruses in cell cultures, whereas significant anti-SARS-CoV-2 activity, documented *in vitro*, are also endorsed by the induction of autophagy *via* the silencing of the PI3K/Akt pathway signalization [147].

For greater clarification of this hypothesis, it must be highlighted that autophagy represents a conserved and a highly complex, multiple-step process, involved in the degradation and recycling of intracellular components [145, 147, 149]. It is induced by starvation and endoplasmic reticulum stress, with the intention of preserving cellular homeostasis [145, 147, 149]. The process of autophagy is activated upon the inhibition of the mammalian target of rapamycin complex 1 (mTORC1), evidenced to be a primary regulator of nutrient signaling [145]. Several upstream regulators of the mTORC1 complex have been demonstrated, whereas the stimulation of PI3K/Akt activates mTORC1 [145, 147], while adenosine monophosphate-activated protein kinase (a sensor of cellular energy levels) inhibits mTORC1 activity [145]. Besides its role in promoting cell-protective processes, under the settings of starvation, recycling, and provision of substrates for novel molecular synthesis [145, 147, 149], autophagy is currently under meticulous scrutiny within the constraints of COVID-19 treatment, namely, for its ability to participate in the secretion of invading pathogens [145, 149, 150]. As a process that promptly responds to any type of stress, it may be foreseen that autophagy participates in viral restriction, through the process of degenerating viral particles, their components, or host proteins used for viral propagation [149, 150], a process already indicated as virophagy [149–151]. There are several lines of evidence showing that SARS-CoV-2 alters the process of autophagy, aiming to enhance viral replication [145–152]. Namely, several viral proteins, including open-reading frame (ORF)3a and nonstructural protein (NSP)6, are directly associated with the initiation of autophagy [152], whereas autophagy seems likely to contribute to the reduction of IL-17- and NLRP3-dependent signaling [149, 153], recognized as important mechanisms in the pathophysiology of COVID-19 [112, 115]. Moreover, viral proteins, to their benefit, negatively regulate the final step of autophagy, causing impaired fusion with the lysosomes, resulting in autophagosomes accumulation, as documented in the lungs of diseased COVID-19 patients [149, 152]. Moreover, coronaviruses modulate PI3K/Akt/mTOR and adenosine monophosphate-activated protein kinase (AMPK) signaling, indicating that SARS-CoV-2 reduced glycolysis and protein translation takes place by reducing the activation of mTORC1 and AMPK [145], in a manner which is directly associated with autophagy. Some research provides the conceptualization that the replication of SARS-CoV-2 directly relates to the autophagy proteins of the host, thereby usurping the autophagy network for its own invasion and replication [145, 150]. Another indication of a link between autophagy and the SARS-CoV-2 replication cycle is the formation of analogous vesicular structures in both cases [145, 147, 149]. The pathophysiology of these vesicles is not completely recognized, although several viral nonstructural proteins (NSP), particularly NSP6 (viral replicase), debilitate the autophagic mechanisms for the generation of the double-membrane vesicles [147].

Within this context, it may be entirely possible that molecules modulating autophagy, at various levels, particularly regarding the multiple step biology, may be anticipated as a therapeutic solution for COVID-19 [145, 147, 149]. As such, compounds that deacetylate regulatory proteins initiate the process of autophagy, followed by *in vitro* inhibition of the SARS-CoV-2 infection (154). This response is likely, at least in part, due to the activation of the NAD^+^-dependent deacetylases (sirtuins), whereas SIRT1, specifically, is evidenced as an mTOR-independent inducer of autophagy [119, 149]. Indeed, both sirtuins (1 and 3), when upregulated by renalase, *in vitro* and *in vivo*, likely induce and inhibit autophagy (and mitophagy), through the upregulation of different downstream signaling, as excessively reviewed elsewhere [119, 154–158]. In line with the aforementioned, mitochondrial dynamics that compromise processes such as autophagy, mitophagy, and enzymes involved in metabolism processes, may be vastly altered during the progression of COVID-19 [159]. In addition, SARS-CoV-2 likely diminishes the supply of ATP to the mitochondria, followed by robust mitochondrial ROS formation, whereas virus propagation is obtained by autophagic alteration, mitochondrial permeability transition pore (MPTP) opening, and NLRP3 inflammasome upregulation [159]. In that context, renalase supplementation, *in vivo*, preserves mitochondrial function (ATP production, mtDNA copy numbers, and mitochondrial dynamics), and rescues mitochondrial network morphology, as evidenced by suppression of mitochondrial fission-related protein (Drp1) alteration, as stated [19]. Drp1-mediated mitochondrial fission is defined as a negative regulator of mitochondrial homeostasis [19], and may be mitigated by renalase upregulation of NAD^+^ levels, followed by activation of SIRT1 [19]. Within that framework, defective mitochondrial fission, regarded as enhanced ERK1/2 signaling and Drp1 alteration, triggers NLRP3 inflammasome assembly, and activates caspase 1 and IL-*β* [160], leading to aberrant inflammation and presumably resulting in a cytokine storm [8, 11, 112, 115]. Renalase's ability to preserve mitochondrial dynamics (by sitruins upregulation), that is seemingly interrupted by SARS-CoV-2, *via* autophagy, mitophagy, and cellular metabolism alteration, supports the concept of renalase's competence in modulating some regulatory points of autophagy, at least by sirtuin activation. The other potentially shared targets of both renalase and autophagy that may be argued in support of treatment for COVID-19 may be MAPKs pathways, particularly ERK/1/2 and JNK signalization. These signaling networks are being evidenced as renalase downstream signals [18, 25, 42, 70], and mediate noncanonical autophagy *via* the regulation of the Bcl-2-interacting protein (beclin 1) [161], and are likely involved in the pathophysiology and prognosis of SARS-CoV-2 infection [162]. In regard to this matter, coronaviruses *in vitro* promote substantial changes in the phosphorylation of ERK1/2 and PI3K/Akt pathways, mechanisms that essentially correspond to renalase pathophysiology [20, 34, 36] and, as such, will be reviewed in detail in the final paragraph. The aforementioned interactions of renalase and proposed responses to the SARS-CoV-2 infection are illustrated in Figure 1 and summarized in detail in Table 4.

Based upon the experimentally acquired knowledge on renalase pathophysiology, it may be hypothesized that renalase, sirtuins, and several types of downstream signaling (e.g., MAPKs), and autophagy share some common aspects, to a point that renalase may be hypothesized as being indirectly implicated as a key player for the control of autophagy. In addition, as vigorously argued throughout the review, renalase has been suggested as an essential player for the processes of inflammation and resolution, in settings that also encompass the process of autophagy [149]. Such perceptions appear captivating considering the current level of expertise of renalase's nature. It may be beneficial to identify strategically important “points of interest” with respect to autophagy modulation and renalase employment within the constraints of the COVID-19 virus. Notwithstanding, regarding the hypothesis of renalase/autophagy axis, substantial gaps in knowledge have yet to be completed and require a great deal of subsequent research.

## 10. Characterization of Renalase as an Antifibrotic Molecule in the Context of COVID-19

Pulmonary fibrosis is a highly heterogeneous pathological process and, among broad etiology factors, may be caused by viral infection. In the majority of patients, this process is refractory to therapeutical treatment; therefore, it is ultimately important to research new agents to prevent the initiation of lung fibrosis and disease progression [15, 16]. The hallmark in the pathogenesis of pulmonary fibrosis represents a combination of ongoing pulmonary lesion and the failure to restore the injury, resulting in a pathological lung fibroproliferative response [15]. In individuals with severe forms and long duration of the disease, as seen in COVID-19-related lung injury, as mentioned previously [56–59], alveolar spaces are left with remarkable and continuous accumulation of inflammatory cells, namely, macrophages, myofibroblasts, fibroblasts, and fibrocytes, whose activation leads to excessive production of extracellular matrix components [15, 16], the phenomenon evidenced in SARS-CoV-2 infection [56–59]. The subsequent pathways contributing to the pulmonary fibroproliferative drive, regardless of the etiology, represent a disturbed balance between profibrotic, namely, TGF-*β* pathway, and antifibrotic mediators, and remarkable differentiation of lung epithelial mesenchymal cells, and other cells into pulmonary fibroblasts [56–59]. The reports that renalase was proven to mitigate the aforesaid pathways, in experimentally induced heart and kidney fibrosis, presume that similar effects may be obtained in pulmonary fibrosis as well. As clearly evidenced, systemic renalase administration by adenovirus significantly alleviates experimentally induced fibrosis of both heart and kidneys, and prevents adverse cardiac remodeling, whereas few plausible pathways of renalase protection were offered [22].

Notably, renalase supplementation reduces inflammatory drive in kidneys through downregulation of proinflammatory cytokine expression, and NADPH oxidase components (gp91^phox^, p47^phox^, and p67^phox^), resulting in lessened glomerular hypertrophy and renal interstitial fibrosis [22]. At the same time, cardiomyocytes hypertrophy and cardiac interstitial fibrosis were reduced, as well as adverse cardiac remodeling, through decreased profibrotic gene expression, and inhibition of phosphorylation of ERK1/2. It should be noted that phosphorylation of ERK1/2, which may be efficiently suppressed by renalase administration, leads to increased oxidative stress and induced fibroblast activation [22]. Finally, exogenous renalase application was once again verified to lessen macrophage accumulation and impede their phenotypic and functional transformation (M1 to M2), presuming that renalase suppresses initial proinflammation *via* inhibition of M1 macrophage polarization and promoting anti-inflammation by enhancing M2-like macrophage polarization, as discussed [22, 42]. As previously argued, the discovery that renalase may interfere with macrophages' phenotypic shifting, directing them towards anti-inflammatory (M2) behavior, deserves particular emphasis in the context of COVID-19 infection. Resident alveolar macrophages, defined as the key cells involved in the cytokine storm-induced fibroproliferative response, promptly after pathogen (SARS-CoV-2) stimulation, shift to the M1 phenotype, releasing abundant proinflammatory and possibly deleterious cytokines (TNF-*α*, IL-1*β*, IL-6, IL-12, IL-18, and IL-23) for neutrophils recruitment [7–12, 163–167]. Therefore, the activation of M1 macrophage phenotype, beside pathogen defense, is evidenced to be significantly responsible for lung tissue deterioration in acute lung injury [165, 166], and apparently in COVID-19-related ARDS. However, the M2 phenotype is greatly involved in lung tissue rescue, through the clearance of apoptotic neutrophils, and cell debris and appropriate release of anti-inflammatory cytokine [9, 166]. This macrophage shifting is proven to be under the control of the classical signaling pathways such as NF-*κβ*, STAT1, STAT3, IFN-*γ* regulatory factor, HIF-1*α*, and activator protein (AP)1 [164, 166]. Among them, HIF-1*α*, NF-*κβ*, and STAT3 are documented to be transcription factors for renalase expression [86]. The hypothesis, whether these are shared mechanisms, mediating and supervising macrophage shifting and renalase transcription, aiming to balance the acute inflammatory burst, needs comprehensive clarification. However, in line with the evidence of renalase's protective role in kidney fibrosis, it may be presumed that renalase controls signaling activation to maintain the balance between the pro- and anti-inflammatory properties of macrophages, aiming to ensure that the immune response is both adequate and proportional. With reference to the aforesaid data, a related research of liver fibrosis indicates that the lack of the renalase gene in knockout mice favors oxidative stress promotion, macrophages accumulation, and TGF-*β*1 expression, finally emerging as liver fibrosis progression [29]. However, in renalase knockout mice experiencing liver fibrosis, the expression of the adhesion G protein-coupled receptor E1 (Adgre1), a mature macrophage marker, was significantly higher compared to the controls, indicating, once again, an apparent renalase-macrophage linking. As confirmed, renalase downregulation seemingly facilitates monocyte-derived macrophages liver tissue accumulation, as well as resident macrophage (Kupffer cells) activation [29]. This hypothesis may be conveniently applied to the lung tissue, as it was demonstrated that monocyte-derived macrophages, attracted to the alveolar lumen after pathogen's stimulation, differentiate to M1 phenotype [9, 166], induce pulmonary fibroproliferation, and persist in the lungs after the resolution of injury. Moreover, it was proposed that their selective targeting may ameliorate lung fibrosis [167], the effects of which hypothetically may be mediated by renalase administration. Based on the current data, modulation of monocytes/macrophages shifting may be a relevant therapeutic strategy against ALI/ARDS [166], and plausibly in the current COVID-19 pandemic. Perhaps it is reasonable to expect that this behavior may be, at least in part, managed *via* renalase supplementation. Albeit, theoretically very appealing, this hypothesis needs comprehensive experimental investigation before clinical employment.

Furthermore, reports confirm that renalase supplementation, following renal injury, hindered the expression of the interstitial matrix components, and mitigated renal fibroproliferative response [20]. It is entirely possible that the proposed mechanism of renalase fibrosis prevention, in the setting of acute kidney injury, may similarly be applied to acute lung injury and COVID-19-related ARDS. Renalase was evidenced to inhibit TGF-*β*1-induced epithelial to mesenchymal transition (EMT) in proximal tubule epithelial cells, a pathological phenomenon that is widely recognized to be similarly involved in the development of lung fibrosis. It has to be outlined that the process of epithelial to mesenchymal transition (EMT) refers to a complex cellular process [168, 169], during which epithelial cells, stimulated by the signals gained from their microenvironment, acquire the phenotype and behavior of myofibroblasts, additionally representing a critical mechanism associated with organ fibrosis. In chronic inflammatory settings, the EMT process is orchestrated *via* signalization upregulated by ROS, proinflammatory cytokines, including HIF-1*α*, TNF-*α*, and TGF-*β*1 [168, 169]. Within this context, TGF-*β* is evidenced to be a key molecular inducer of the EMT molecular programming, through the activation of PI3K/Akt and MAPKs signalization [169]. In the milieu of unrestrained inflammatory response, which likely occurs during severe COVID-19 cases, extensive EMT provides the excessive formation of myofibroblasts, generating progressive fibrosis. The inhibition of the TGF-*β*1-induced EMT is obtained owing to renalase's capacity (*in vitro*) to lessen the expression of *α*-smooth muscle actin (*α*-SMA), fibronectin, and collagens (I and III), while the expression of E-cadherin was restored. However, renalase was, *in vitro*, confirmed to initiate the inhibition of TGF-*β*-mediated upregulation of *α*-SMA and downregulation of E-cadherin. In this context, TGF-*β*1-stimulated cells obtained higher levels of phospho-ERK1/2, which were reversed following adenoviral renalase supplementation. In a similar manner, in rats with diabetic nephrosclerosis [34], renalase supplementation significantly attenuated high glucose-induced profibrotic gene expression, and p21 expression *via* the silencing of ERK1/2 signals, whereas a more recent study evidenced that recombinant renalase significantly alleviated pressure overload-induced fibrosis in the heart, being highly associated with p38 and ERK1/2 signaling [36].

Therefore, clear evidence exists that renalase mitigates renal interstitial fibrosis through the inhibition of the ERK1/2 signaling pathways [20]. The described pathophysiology is worthy of comparison and essentially corresponds to the proposed pathogenesis of pulmonary fibrosis following SARS-CoV-2 infection. Mounting reports support the biology of myofibroblasts being the cells committed to produce denser and more disorganized components of extracellular matrix, originating from alveolar fibroblasts that migrated to the site of the injury and subsequently proliferated [9, 16, 166, 167]. Owing to a *α*-SMA actin presence (that may be efficiently *in vitro* suppressed by renalase) [20], these cells may significantly reorganize, resulting in irreversible rearrangement of collagen fibrils, that is one of the key characteristics of fibrogenesis [9, 16, 167]. Moreover, myofibroblasts found in the lung tissue, similar to that in the kidneys [170], may equally originate from epithelial- and endothelial-mesenchymal transition, where alveolar epithelial cells lose their markers (surfactant proteins, mucin, and adhesion molecules) and cytoskeletal proteins, being replaced with mesenchymal cell markers (*α*-SMA, vimetin, and fibronectin) [9, 171–173], and under the supervision of TGF-*β*1 pathway [173]. Based on these premises, it is quite reasonable to believe that the mechanism of renalase protection, as described in the kidneys, may be applied in the lungs in a corresponding manner, with the same intention of impeding the expression of *α*-SMA, and fibronectin, and the suppression of alveolar epithelial to mesenchymal transition, through the inhibition of the ERK1/2 pathway. If proven that renalase exerts the same effects in lungs, likewise kidneys, it may remarkably improve the outcome of ARDS patients, regardless of its underlying cause. Within the context of renalase's remote roles, additional mechanisms of renalase's antifibrotic traits were identified [21], providing an expertise that may be expanded to the pathophysiology of COVID-related ARDS. Numerous researches reported that increased ROS production represents a common mechanism of organ injury and that their formation may be considered a precursor of fibrogenesis. Nevertheless, *in vitro* studies confirmed that ROS demonstrate a pivotal role in TGF-*β*-induced EMT, through the activation of MAPK (non-Smad) pathways in renal epithelial cells, leading to renal interstitial fibrosis [174], and that pharmacological inhibition of ROS generation and inactivation of PI3K/Akt and MAPK pathways alleviate TGF-*β*1-induced EMT in bronchial epithelial cells, resulting in minimized pulmonary fibrosis [175]. The particular role for renalase is to directly block the ERK1/2 and p38 pathways, as already discussed, and may be regarded in this context with the most recent recognition of pathophysiology of SARS-CoV-2 pathogen to promote p38 signaling activity and cell cycle arrest [6, 162]. In line with that, downregulation of renalase signaling is associated with sustained activation of p38, and *vice versa* [77, 176]. However, it was recently reported that during COVID-19, inhibition of p38 MAPK resulted in suppressed cytokine production, and impaired viral replication by a still unknown mechanism, suggesting that p38 inhibition may target multiple mechanisms related to COVID-19's pathogenesis [6, 14]. These observations led to the outset of current clinical trials investigating small molecules aiming to inhibit those pathways (p38 MAPK and the p38 MAPK-MAPKAPK2 signaling complex), having the potential of being anti-COVID-19 drugs [177]. The aforementioned pathways are depicted in Figure 1 and comprehensively summarized in Table 5.

The final consideration relating to the possible “duality” of renalase's actions implies that renalase most likely functions in a context- or time-dependent signaling (i.e., state of the target cell, experimental conditions, time-dependent, or different tissue), employing its impressive ability for context-related adjustments of the MAPK pathways [18, 20, 25, 34, 36]. This knowledge upholds the appealing possibility that renalase administration in COVID-19, by mediation, or supervision of MAPKs signalization, may be as efficient as the protocols directed against one particular cytokine. A summary of current experimental data regarding renalase antifibrotic traits is given in Table 5, and the proposed signaling arms that may be targeted by renalase, in the context of fibroproliferation, are depicted in Figure 1.

## 11. Conclusion

The COVID-19 pandemic represents a gargantuan health care, social, economic, and political burden in general, while its long-term consequences will seriously continue to challenge the entire community. For these reasons, all identified risk factors and pathophysiological processes of SARS-CoV-2-related disease, which are feasible for the prevention and treatment, should be addressed in a timely manner. Accordingly, the evolving anti-inflammatory and antifibrotic therapy for severe COVID-19 and hindering post-COVID-19 fibrosis development should be comprehensively investigated.

A complex interaction exists between renalase, inflammation, oxidative stress, apoptosis, and fibroproliferation, which is emphasized by the experimentally evidenced renalase's remarkable actions, presumably in order to mitigate adverse fibroproliferative outcome. Effective anti-COVID-19 approach may take advantage of renalase's pleitropic effects including: lessening of neutrophils and macrophages accumulation, and macrophage phenotype shifting (M1/M2), a decrease of the proinflammatory cytokines (TNF-*α*, IL-6, MCP-1, MIP-2, and TGF-*β*1) release, and ROS generation, an increase in antiapoptotic factors (Bcl-2), and prevention of caspase-3 activation, inflammasome silencing, increasing the intracellular concentration of NAD^+^, thereby sirtuins (1 and 3) activation, hence preserving mitochondrial biogenesis, and dynamics, epithelial to mesenchymal transition suppression, a decrease in the profibrotic markers expression (*α*-SMA, collagen I,III, TIMP-1, and fibronectin), and interference with MAPKs signaling network. This review, based on the mounting quantities of published data, supports the hypothesis that exogenous renalase administration, presumably coupled with other COVID-19 protocol-approved drugs, may prove beneficial during COVID-19-related lung injury. Albeit that renalase expression was not investigated in pulmonary tissue, so far, its sustained plasma concentration, systemic behavior and multifaceted nature, various tissue distribution, similar triggering, and distinctive roles provide a substantial rationale that its protective manner may be obtained for the lungs, in a similar manner as evidenced in other organs.

Novel therapies for COVID-19 seek to exploit renalase's multiple and distinctive cytoprotective mechanisms; therefore, this review should be acknowledged as the thorough groundwork for subsequent research of renalase's employment in the experimental models of SARS-CoV-2 infection. Finally, it is of utter importance for the entire scientific society to comprehensively explore and conjoin all available knowledge to win the battle against this devastating pandemic.

## Figures and Tables

**Table 1:** The assessment of renalase in COVID-19 hospitalized patients.

Study model	The aim of the study	Study evidence	Health perspective	Ref
COVID-19 (hospitalized patients)	The determination of renalase and proinflammatory biomarkers in hospitalized patients (51) and its association with disease severity and survival.	Renalase concentration negatively correlates with inflammatory markers, and its low concentration is associated with more severe COVID-19, and with worse survival among COVID-19 patients.	A biomarker for identification of severe COVID-19 and a disease prognostic factor.	90
COVID-19 (hospitalized patients)	The determination of renalase and cytokines in hospitalized patients (458), and its association with intubation or death within 180 days, overall mortality, ICU admission, use of vasopressors, and CPR.	Decreased renalase is associated with higher hypoxia, increased ICU admission, a longer length of stay, use of vasopressors, and CPR rate, and is independently associated with intubation and higher mortality in hospitalized COVID-19 patients.	A biomarker for COVID-19 progression and a disease prognostic factor.	91
COVID-19 (hospitalized patients)	The association of asymptomatic infection and symptomatic COVID-19 with renalase, and specific biochemical, renal, and immune parameters in hospitalized patients (58).	Renalase is progressively increased after hospital admission, and its concentration is related to the activation of the immune system.	A biomarker for disease monitoring.	92

COVID-19: coronavirus disease 2019; ICU: intensive care unit; CPR: cardiopulmonary resuscitations.

**Table 2:** Antioxidation, anti-inflammation, antinecrosis, and antiapoptosis effects of renalase based upon current experimental evidences.

Study model	The aim of the study	Study evidence	Health perspective	Ref
Acute kidney injury	The assessment of renalase's reno-protective properties in mice with cisplatin-induced AKI (*in vivo*), and in HK-2 cells (*in vitro*), against oxidant and cisplatin damage.	Renalase deficiency results in significant tubular necrosis, apoptosis, and macrophage infiltration, whereas renalase treatment reduces caspase-3 activation and increases Bcl-2 expression.	Therapy for AKI	18
Fatty liver IR injury	The assessment of renalase effects on oxidative stress, mitochondrial function, necrosis, apoptosis, and enzymes dynamics, in fatty liver IR injury, in mice (*in vivo*), and in HepG2 cells (*in vitro*).	Renalase mitigates fatty liver IR injury *via* attenuation of oxidative stress, and apoptosis, by suppressing cyto C liberation, and Bax expression, and elevating Bcl-2 expression, and mitochondrial damage through SIRT1 activation.	Therapy for liver IR injury	19
Chronic kidney disease	The role of renalase in the progression of cardiorenal syndrome in rats after subtotal nephrectomy (*in vivo*)	Renalase reduces oxidative stress, and macrophage infiltration, activation and polarization, and proinflammatory cytokines (TNF-*α*, IL-6, and MCP-1), and NADPH oxidase components (gp91^phox^, p47^phox^, and p67^phox^) expression, including the significant cardiovascular and renal protection.	Cardiorenal protection in chronic kidney disease patients	22
Renal IR injury (ischemic AKI)	The outcome of renal IR injury in renalase-deficient mice (*in vivo*) and the response of renalase supplementation.	Renalase ameliorates renal necrosis, apoptosis, neutrophil and macrophage infiltration, and its deficiency increases proinflammatory gene expression (TNF-*α*, MCP-1, and MIP-2).	The prevention and treatment of AKI	23
Renalase-deficient mice, and isolated hearts	The association of renalase deficiency and cardiovascular complications in knockout mice (*in vivo*) and the effects of renalase reperfusion on ischemic cardiac damage in isolated hearts (*in vitro*).	The renalase deficiency results in an increased degree of myocardial necrosis, elevated blood pressure, heart rate, and left ventricular hypertrophy. Renalase reperfusion reduces the size of the infarct.	The improvement of cardiovascular outcome in chronic kidney disease patients	24
Acute pancreatitis	The renalase effects in rats with cerulein-induced pancreatic injury (*in vivo*), and in isolated pancreatic lobules (*in vitro*), on cell injury, and the outcome in renalase deletion mice and renalase administration.	Renalase pretreatment decreases pancreatitis responses, and its administration results in a significant decrease in neutrophil infiltration, less morphologic edema, and fewer vacuoles.	Treatment for acute pancreatitis	26
Contrast-induced nephropathy	The effects of renalase pretreatment in rats with loversol-induced CIN (*in vivo*) and in HK-2 cells (*in vitro*).	Renalase preconditioning inhibits renal apoptosis and caspase-3 activity, reduces oxidative stress (decreases MDA and increases SOD levels), and decreases TNF-*α* and MCP-1 expression and macrophage infiltration.	Prevention against contrast media-induced AKI	27
Myocardial IR injury	The effects of renalase in rats (*in vivo*) subjected to MIRI, and their outcome after renalase treatment.	Renalase deficiency promotes myocardial cells necrosis and apoptosis, whereas pretreatment with renalase reduces this response.	IR myocardial injury protection.	28
Nonalcoholic steatohepatitis	The effects of renalase deficiency in mice with nonalcoholic steatohepatitis (*in vivo*) on oxidative stress, inflammation, liver function, and fibrosis.	The deficiency of the renalase gene enhances the progression of oxidative stress and inflammation and promotes TGF-*β* upregulation and liver function deterioration in nonalcoholic steatohepatitis.	Antioxidative protection in liver fibrosis	29
Cisplatin-induced AKI	The renalase effects on ROS generation, apoptosis, mitochondrial dynamics, and renal function, in cisplatin-induced kidney injury mice (*in vivo*), and in HK-2 cells (*in vitro*).	Renalase protects against cisplatin-induced AKI by inhibiting oxidative stress and improving mitochondrial function in a SIRT3-dependent manner.	Reno-protection in patients treated with cisplatin.	30
HK-2 cells	The assessment of renalase expression in HK-2 cells (*in vitro*), as a response to epinephrine.	The renalase secretion is evoked by epinephrine *via α*-adrenoceptor/NF-*κβ* pathways in renal proximal tubular epithelial cells.	Treatment of hypertension and CKD	31
Contrast-induced nephropathy	The renalase effects in the limb IPC in CIN rats (*in vivo*) and in HK-2 cells (*in vitro*).	The renalase expression is induced by TNF-*α*, through activation of NF-*κβ* signaling, and protects from oxidative stress, apoptosis, and inflammation.	Renal protection in CIN	32
Cisplatin-induced CKD	The outcome of kidney-targeted delivery of renalase agonist in mice with CKD (*in vivo*) on kidney morphology.	Kidney-targeted agonist of renalase ameliorates the degree of inflammation, necrosis, and myofibroblasts and prevents cisplatin-induced kidney damage.	Reno-protection in patients receiving cisplatin therapy	37
Treadmill exercise	The effects of different acute exercise intensities on renalase expression in mice (*in vivo*).	Renalase expression in skeletal muscles increases after acute exercise, as a response to exercise-induced oxidative stress.	Antioxidative protection	43
Hepatic IR injury	The response of renalase to oxidative stress in mice with liver IR injury (*in vivo*) and in HepG2 cells (*in vitro*).	Renalase is responsive and sensitive to oxidative injury and may be suppressed by antioxidants treatment.	Antioxidative liver protection and a biomarker for the liver IR injury	72
Small intestine and Caco-2 oxidative injury	The effects of oxidative stress on renalase expression and localization in mice with fasting-induced oxidative stress (*in vivo*) and in Caco-2 cells (*in vitro*).	Renalase expression in small intestine is upregulated *via* NF-*κβ* p65 activation and is induced by oxidative stress.	Antioxidative intestinal protection	73

AKI: acute kidney injury; HK-2 cells: human proximal renal tubular epithelial cells; IR: ischemia/reperfusion; HEPG2 cells: hepatocellular carcinoma; SIRT1: sirtuin 1; NADPH: nicotinamide adenine dinucleotide phosphate oxidase; MDA: malondialdehyde; SOD: superoxide dismutase; MCP-1: monocyte chemoattractive protein 1; MIRI: myocardial/ischemia reperfusion injury; TGF-*β*: transforming growth factor-*β*; ROS: reactive oxygen species; CKD: chronic kidney disease; CIN: contrast-induced nephropathy; IPC: *ischemic* preconditioning; HepG2: human hepatocellular carcinoma cell line; Caco-2: colon carcinoma-2 cell line.

**Table 3:** The associations of renalase and macrophage infiltration based upon current experimental studies.

Study model	The aim of the study	Study evidence	Health perspective	Ref
Cisplatin-induced AKI	The effects of renalase deficiency in knockout mice with cisplatin-induced AKI (*in vivo*) and the outcome of renalase administration in HK-2 against cisplatin and oxidant damage (*in vitro*).	The lack of the renalase gene results in a significant macrophage infiltration, severe acute tubular necrosis, and apoptosis, whereas renalase administration restores cells viability *via* ERK1/2, p38 and PI3K/Akt activation, and JNK downregulation.	The prevention and treatment of AKI in patients treated with cisplatin	18
Cardiorenal syndrome after subtotal nephrectomy	The outcome of renalase administration in rats with subtotal nephrectomy (*in vivo*) on parameters of renal injury and cardiac remodeling.	Renalase treatment inhibits total macrophage infiltration, particularly M1-like phenotype (CD86), and activation and polarization; decreases the expression of MCP-1, TNF-*α*, IL-6, and NADPH oxidase components; reduces expression of collagen I, collagen III, TGF-*β*1, and TIMP-1; and increases expression of MMP-1, including significant amelioration of cardiac and renal injury.	Cardiorenal protection in CKD patients	22
Renal IR injury (ischemic AKI)	The effects of renalase deficiency in mice with ischemic AKI (*in vivo*) and the assessment of the outcome of renalase supplementation.	Renalase administration ameliorates macrophage and neutrophil infiltration, renal necrosis, and apoptosis, whereas the lack of renalase leads to increased TNF-ἀ, MCP-1, and MIP-2 expression.	Biomarker for ischemic AKI, prevention, and treatment of AKI	23
Acute pancreatitis	The effects of renalase in rats with cerulein-induced pancreatic injury (*in vivo*), and in isolated pancreatic lobules (*in vitro*), on cell injury and histological changes, including the outcome in mice with genetic deletion of renalase.	Renalase reduces macrophage and neutrophil infiltration and alleviates pancreatic acinar cell injury through activation of a PMCA4b.	Treatment for acute pancreatitis	26
Contrast-induced nephropathy	The effects of renalase pretreatment in rats with loversol-induced CIN (*in vivo)* and in HK-2 cells (*in vitro*).	Renalase pretreatment decreases macrophages infiltration and renal MCP-1 and TNF-*α* levels and mitigates the decline of renal function, tubular necrosis, oxidative stress, and apoptosis.	Renal protection in patients subjected to CIN	27
Nonalcoholic steatohepatitis	The effects of the renalase deficiency in renalase knockout mice with nonalcoholic steatohepatitis (*in vivo*) on inflammation, liver function, oxidative stress, and fibrosis.	The deficiency of the renalase gene enhances the macrophage infiltration (increased Adgre1 expression), the progression of oxidative stress, and TGF-*β*1 upregulation, and liver function deterioration in nonalcoholic steatohepatitis.	The mitigation of the progression of liver fibrosis	29
Contrast-induced nephropathy	The assessment of renalase effects in the limb IPC-induced reno-protection in CIN rats (*in vivo*) and in HK-2 cells (*in vitro*).	The knocking down of renalase exacerbates renal macrophage (CD68) infiltration, renal function decline, tubular cell apoptosis, and oxidative stress in CIN rats, whereas limb IPC-mediated reno-protection is dependent on renalase upregulation *via* TNF-*α*/NF-*κβ* signaling activation.	Renal protection in CIN	32
Diabetic nephropathy	The assessment of the renoprotective effects of renalase in DN in renalase knockout mice (*in vivo*), renalase's expression in human kidney biopsies with DN, and the effects of renalase on high glucose-induced mesangial cells (*in vitro*).	Renalase ameliorates renal inflammation, and mesangial hypertrophy, and attenuates profibrotic gene expression and p21 expression through the inhibition of the ERK1/2 pathway.	Protection against the progression of diabetic nephropathy	34
Cisplatin-induced chronic kidney disease	The outcome of kidney-targeted delivery of renalase agonist in mice with CKD induced by cisplatin (*in vivo*) on kidney function and morphology.	Kidney-targeted agonist of renalase ameliorates the degree of inflammatory macrophages, necrosis, and myofibroblasts and prevents cisplatin-induced kidney damage.	Reno-protection in patients receiving cisplatin therapy	37
Melanoma cell lines (human and mouse metastatic melanoma) and tumor samples (human skin and mice)	The assessment of renalase expression in primary melanomas and CD163^+^ tumor-associated macrophages (*in vivo* and *in vitro*) and renalase's correlation with disease-specific survival.	Dysregulated renalase signaling promotes macrophage polarization towards CD163^+^ (M2-like) phenotype, whereas renalase upregulation predominantly occurs in M2-like (CD163^+^) macrophages. Inhibition of renalase signaling increases the ratio of M1- to M2-like cells and decreases renalase secretion by CD163^+^ macrophages. The PMCA4b mediates renalase-dependent ERK1/2 phosphorylation in macrophages.	Prognostic marker in melanoma and antirenalase therapy for the treatment of malignant melanoma	177

AKI: acute kidney injury; HK-2 cells: human proximal renal tubular epithelial cells; ERK1/2: extracellular regulated protein kinases 1 and 2; PI3K/Akt: phosphatidylinositol 3-kinase/protein kinase B; JNK: c-Jun N-terminal kinase; MCP-1: *monocyte chemoattractant protein-1*; NADPH: nicotinamide adenine dinucleotide phosphate oxidase; TGF-*β*1: transforming growth factor-*β*1; TIMP-1: tissue inhibitor of metalloprotease-1; MMP-1: *matrix metalloproteinase 1*; CKD: *chronic kidney disease*; IR; ischemia reperfusion; MIP-2: macrophage-inflammatory protein 2; PMCA4: plasma membrane calcium ATPase 4b; CIN: contrast-induced nephropathy; Adgre 1: adhesion G protein-coupled receptor E1; IPC: ischemic preconditioning; DN: diabetic nephropathy.

**Table 4:** The associations of renalase and sirtuins based upon current experimental evidence.

Study model	The aim of the study	Study evidence	Health perspective	Ref
Fatty liver IR injury	The assessment of renalase's effects on liver necrosis, apoptosis, enzymes, oxidative stress, and mitochondrial function, including renalase's expression, role, and mechanisms in fatty liver IR injury, in mice (*in vivo*), and in HepG2 (*in vitro*).	Renalase mitigates liver IR injury *via* ROS generation amelioration and improves mitochondrial function through activating SIRT1, by upregulation of NAD^+^ levels, whereas renalase downregulation is transcriptionally mediated by STAT3.	Liver protection from IR in NAFLD patients.	19
Cisplatin-induced AKI	The effects of renalase on cell viability, renal function, apoptosis, ROS generation, and mitochondrial dynamics in cisplatin-induced kidney injury mice (*in vivo*) and HK-2 cells (*in vitro*).	Renalase ameliorates kidney injury by mitochondrial dynamics promotion, and by inhibiting ROS production, in a SIRT3-dependent manner.	Reno-protection in patients treated with cisplatin.	30

IR: ischemia/reperfusion; HepG2: human hepatocellular carcinoma cell line; ROS: reactive oxygen species: SIRT: sirtuin; NAD: nicotinamide adenine dinucleotide; NAFLD: nonalcoholic fatty liver disease; STAT3: signal transducer and activator of transcription; AKI: acute kidney injury; HK-2 cells: human proximal renal tubular epithelial cells.

**Table 5:** Antifibrosis effects of renalase based upon current experimental evidence.

Study model	The aim of the study	Study evidence	Health perspective	Ref
Cisplatin-induced AKI	The effects of renalase deficiency in knockout mice with cisplatin-induced AKI (*in vivo*), and the outcome of renalase administration in HK-2 against cisplatin and oxidant damage (*in vitro*).	Renalase deficiency results in severe acute tubular necrosis, apoptosis, and macrophage infiltration, and renalase administration restores cells viability *via* ERK1/2, p38 and PI3K/Akt activation, and JNK downregulation.	The prevention and treatment of AKI in patients treated with cisplatin	18
Unilateral ureteral obstruction	The renalase's efficiency in rats with complete unilateral ureteral obstruction (*in vivo*), and renalase effects on TGF-*β*1-induced EMT in HK-2 cells (*in vitro*).	Renalase restores the E-cadherin expression and abolishes *α*-SMA, fibronectin, and collagen-I expression and alleviates renal interstitial fibrosis by inhibiting the tubular EMT *via* ERK1/2 signaling pathways inhibition.	Renoprotection in renal interstitial fibrosis and mitigation of CKD	20
Unilateral ureteral obstruction	The association of renalase and oxidative stress in UUO rats (*in vivo*) and with EMT in HK-2 (*in vitro*).	Renalase administration abolishes oxidative stress-induced *α*-SMA, fibronectin, and collagen I and III expression and restores E-cadherin expression.	Antifibrotic renal protection in CKD.	21
Cardiorenal syndrome after subtotal nephrectomy	The effects of renalase administration in rats with subtotal nephrectomy (*in vivo*) on parameters of renal injury and cardiac remodeling.	Renalase attenuates proteinuria, glomerular hypertrophy, and interstitial fibrosis; decreases profibrotic genes expression, proinflammatory cytokines, and NADPH oxidase components; alleviates hypertension, cardiomyocytes hypertrophy, and cardiac interstitial fibrosis; and prevents cardiac remodeling by profibrotic genes suppression and phosphorylation of ERK1/2.	Cardiovascular and renal protection in patients with CKD	22
Nonalcoholic steatohepatitis	The effects of renalase deficiency in mice with nonalcoholic steatohepatitis (*in vivo*) on liver function, inflammation, oxidative stress, and fibrosis.	The lack of renalase enhances the progression of oxidative stress, macrophage infiltration, TGF-*β* expression, and liver function decline in nonalcoholic steatohepatitis.	The suppression of liver fibrosis progression	29
Diabetic nephropathy	The renoprotective effects of renalase in DN in renalase knockout mice (*in vivo*), renalase's expression in human kidney biopsies with DN, and the effects of renalase on high glucose-induced mesangial cells (*in vitro*).	Renalase ameliorates mesangial hypertrophy and renal inflammation and attenuates profibrotic gene expression and p21 expression through the inhibition of ERK1/2 pathway.	Protection against the progression of diabetic nephropathy	34
Transverse aortic constriction-induced HF	The renalase's role in the pressure overload-induced hypertrophic response in rats (*in vivo*), and in the noradrenaline-induced hypertrophic response (*in vitro).*	Renalase improves pressure overload-induced heart failure by regulating p38 and ERK1/2 signaling network.	A biomarker of cardiac hypertrophy and a HF therapy	36
Chronic kidney disease	The effects of renalase administration in 5/6 nephrectomized rats (*in vivo*) on the severity of cardiovascular disease.	Renalase administration reduces left ventricular hydro-xyproline concentration, as a measure of fibrosis, and the degree of cardiac hypertrophy and cardiac dysfunction.	Cardiovascular protection in patients with chronic kidney disease	38

AKI: acute kidney injury; HK-2 cells: human proximal renal tubular epithelial cells; ERK1/2: extracellular regulated protein kinases 1 and 2; PI3K/Akt: phosphatidylinositol 3-kinase/protein kinase B; JNK: c-Jun N-terminal kinase; TGF-*β*1: transforming growth factor-*β*1; EMT: epithelial-mesenchymal transition; *α*-SMA: *α*-smooth muscle actin; CKD: chronic kidney disease; UUO: unilateral ureteral obstruction; NADPH: nicotinamide adenine dinucleotide phosphate oxidase; DN: diabetic nephropathy; HF: heart failure.

## Data Availability

The literature used to support the findings of this review is listed within the article (bibliography).
